# Impaired saccadic eye movements in multiple sclerosis are related to altered functional connectivity of the oculomotor brain network

**DOI:** 10.1016/j.nicl.2021.102848

**Published:** 2021-10-04

**Authors:** J.A. Nij Bijvank, E.M.M. Strijbis, I.M. Nauta, S.D. Kulik, L.J. Balk, C.J. Stam, A. Hillebrand, J.J.G. Geurts, B.M.J. Uitdehaag, L.J. van Rijn, A. Petzold, M.M. Schoonheim

**Affiliations:** a Amsterdam UMC, Vrije Universiteit Amsterdam, Department of Neurology, MS Center and Neuro-ophthalmology Expertise Center, Amsterdam Neuroscience, Amsterdam, the Netherlands; b Amsterdam UMC, Vrije Universiteit Amsterdam, Department of Ophthalmology, Neuro-ophthalmology Expertise Center, Amsterdam Neuroscience, Amsterdam, the Netherlands; c Amsterdam UMC, Vrije Universiteit Amsterdam, Department of Anatomy and Neurosciences, Amsterdam, the Netherlands; d Amsterdam UMC, Vrije Universiteit Amsterdam, Department of Clinical Neurophysiology and Magnetoencephalography Center, Amsterdam Neuroscience, Amsterdam, the Netherlands; e Onze Lieve Vrouwe Gasthuis, Department of Ophthalmology, Amsterdam, the Netherlands; f Moorfields Eye Hospital, The National Hospital for Neurology and Neurosurgery and the UCL Queen Square Institute of Neurology, London, United Kingdom

**Keywords:** Multiple sclerosis, Neuro-ophthalmology, Magnetoencephalography, Eye movement, Brain network function, Biomarkers, MS, multiple sclerosis, FC, functional connectivity, MEG, magnetoencephalography, INO, internuclear ophthalmoplegia, EDSS, Expanded Disability Status Scale, Pv/Am, peak velocity divided by amplitude, BNA, Brainnetome Network Atlas, AECc, corrected Amplitude Envelope Correlation

## Abstract

**Background:**

Impaired eye movements in multiple sclerosis (MS) are common and could represent a non-invasive and accurate measure of (dys)functioning of interconnected areas within the complex brain network. The aim of this study was to test whether altered saccadic eye movements are related to changes in functional connectivity (FC) in patients with MS.

**Methods:**

Cross-sectional eye movement (pro-saccades and anti-saccades) and magnetoencephalography (MEG) data from the Amsterdam MS cohort were included from 176 MS patients and 33 healthy controls. FC was calculated between all regions of the Brainnetome atlas in six conventional frequency bands. Cognitive function and disability were evaluated by previously validated measures. The relationships between saccadic parameters and both FC and clinical scores in MS patients were analysed using multivariate linear regression models.

**Results:**

In MS pro- and anti-saccades were abnormal compared to healthy controls A relationship of saccadic eye movements was found with FC of the oculomotor network, which was stronger for regional than global FC. In general, abnormal eye movements were related to higher delta and theta FC but lower beta FC. Strongest associations were found for pro-saccadic latency and FC of the precuneus (beta band β = -0.23, *p* = .006), peak velocity and FC of the parietal eye field (theta band β = -0.25, *p* = .005) and gain and FC of the inferior frontal eye field (theta band β = -0.25, *p* = .003). Pro-saccadic latency was also strongly associated with disability scores and cognitive dysfunction.

**Conclusions:**

Impaired saccadic eye movements were related to functional connectivity of the oculomotor network and clinical performance in MS. This study also showed that, in addition to global network connectivity, studying regional changes in MEG studies could yield stronger correlations.

## Highlights

• Impaired eye movements in multiple sclerosis (MS) and functional connectivity (FC)
• Eye movements related to altered FC of the oculomotor brain network.
• Lower (beta band) and higher (theta/delta band) FC related to abnormal eye movements.
• Regional changes were more informative than whole-network measures.
• Eye movement parameters also related to disability and cognitive dysfunction.

## 1 Introduction

In many neurological diseases, the shift in focus from studying focal pathology to functional brain networks has increased our understanding of the etiology of clinical dysfunction, which is especially relevant in multiple sclerosis (MS). (Mollison et al., 2017, Schoonheim et al., 2014, Schoonheim et al., 2015) Functional brain network inefficiency resulting from accumulating structural damage varies between individuals, but eventually leads to clinical and cognitive decline in MS. (Schoonheim et al., 2015) Network dysfunction has mainly been studied with functional (f)MRI in MS, a measure of brain function based on levels of oxygenated blood. Magneto-encephalography (MEG) is a neurophysiological modality that directly measures neuronal activity with high temporal resolution. Recently, studies have shown that MEG is able to detect clinically relevant disruptions in functional networks in MS, potentially with higher sensitivity than fMRI. (Nauta et al., 2020, Tewarie et al., 2015, Sjøgård et al., 2021) These insights have underlined the importance of brain network changes in MS, but network evaluation in routine clinical practice remains impossible. Therefore, the search for easily measured and objective clinical outcome measures that can reflect abnormalities of the functional network is remains ongoing. (van Munster and Uitdehaag, 2017) Eye movement measurement is a strong candidate for such a marker of network dysfunction.

Eye movements are the fastest movements of the human body with highly consistent patterns of movement. (Leigh and Zee, 2015) Eye movement abnormalities are common is MS and clinically relevant due their disabling nature in daily life. (Nij Bijvank et al., 2019, Jasse et al., 2013) Recently, it has been shown that they relate to general disability, cognitive function and neurodegeneration in MS. (Nij Bijvank et al., 2019, Nij Bijvank et al., 2020, Fielding et al., 2015, Kincses et al., 2019) Recent methodological advances now enable a precise and non-invasive way of detecting (subclinical) eye movement deficits using high-frequency infrared oculography. (Leigh and Zee, 2015, Nij Bijvank et al., 2018, Frohman et al., 2003) With this technique the well-known pathology of internuclear ophthalmoplegia (INO) can be readily diagnosed. (Nij Bijvank et al., 2019, Frohman et al., 2003) In addition, precise quantification of more subtle abnormalities has become feasible. (Nij Bijvank et al., 2020, Sheehy et al., 2020, Nij Bijvank et al., 2019) Importantly, eye movements are generated by a wide-spread and coordinated functional network. This network integrates sensory, motivational, executive and motor information, which is also extensively involved in cognitive function. (Leigh and Zee, 2015, Fielding et al., 2015, Coiner et al., 2019)

Despite these theoretical arguments, the oculomotor network and the associations with eye movement abnormalities have not yet been studied in MS. We hypothesize that eye movements are more strongly related to functional connectivity of this oculomotor network than to whole brain functional connectivity. We aim to identify which eye movement measures are most indicative of this network and evaluate if the network as a whole or regional changes are most relevant. In addition, we verified whether and which eye movement measures also reflect clinical and cognitive function.

## 2 Materials and Methods

### 2.1 Study design and patient population

For this observational cross-sectional study, MS patients and healthy controls were included from the Amsterdam MS cohort, as previously described. (Nij Bijvank et al., 2019, Nij Bijvank et al., 2019) This study was approved by the Medical Ethical Committee on Human research of the Amsterdam UMC and followed the tenets of the Declaration of Helsinki. Written informed consent was obtained from all participants before study inclusion. Included patients were diagnosed with clinically definite MS according to the revised McDonald criteria, (Polman et al., 2011) and subdivided into relapsing-remitting, secondary progressive and primary progressive MS. (Lublin and Reingold, 1996) The Expanded Disability Status Scale (EDSS) score was used to assess the level of disability of MS patients. (Kurtzke, 1983) All assessments and data collection (clinical, infrared oculography, magnetic resonance imaging (MRI), magnetoencephalography (MEG) and neuropsychological evaluation), were performed on the same day and in the same order, as previously described. (Nij Bijvank et al., 2019, Nij Bijvank et al., 2019)

### 2.2 Infrared oculography

Eye movements were measured and analysed using our standardised infrared video-oculography protocol, the DEMoNS protocol. (Nij Bijvank et al., 2018) In brief, eye movements were measured binocularly with the Eyelink 1000 Plus eye tracker at 1000 Hz during a pro-saccadic and an anti-saccadic task as described previously. (Nij Bijvank et al., 2020, Nij Bijvank et al., 2018)

An off-line analysis was performed in Matlab (Mathworks, Inc., Natick, MA; version 2017b) to automatically calculate movement parameters, (Nij Bijvank et al., 2018) including latency, peak velocity, and gain of the centrifugal saccade. (Nij Bijvank et al., 2018, Antoniades et al., 2013) In the pro-saccadic task only, a main sequence relationship was calculated by dividing the peak velocity by the saccadic amplitude (Pv/Am). (Leigh and Zee, 2015, Nij Bijvank et al., 2020, Nij Bijvank et al., 2018) For the anti-saccadic task a set of three additional parameters was calculated, namely: (1) the proportion of errors (first saccade in the same direction as the target), (2) the latency of a correction saccade after an incorrect response and (3) the spatial error of the final eye position after the anti-saccade (before reappearance of the target). (Nij Bijvank et al., 2020, Nij Bijvank et al., 2018) Parameters were averaged over the left and right eye. Presence of internuclear ophthalmoplegia (INO) was determined using Versional Dysconjugacy Index based thresholds. (Nij Bijvank et al., 2019). A higher value of latencies and errors, and a lower value of peak velocity and gain were interpreted as worse eye movement performance. (Nij Bijvank et al., 2020)

### 2.3 Magnetoencephalography (MEG)

Resting-state MEG measurements were pre-processed using a standardized procedure. (Nauta et al., 2020, Hillebrand et al., 2016) In short, MEG data were acquired using a 306-channel whole head MEG system (Elekta Neuromag Oy, Helsinki, Finland), situated in a magnetically shielded room. Eyes-closed resting state measurements were performed (5 min) at 1250 Hz. MEG data were visually inspected to discard malfunctioning channels and the temporal extension of Signal Space Separation (tSSS) was used to remove artefacts. (Taulu and Simola, 2006) Source-localized MEG data were constructed for 224 cortical and thalamic regions (listed in Supplementary Table 1) of the Brainnetome Network Atlas (BNA) (Fan et al., 2016) using an atlas-based beamformer. (Hillebrand et al., 2012) An advantage of the BNA atlas is that it is created using a connectivity-based parcellation applying multimodal connectivity information. (Fan et al., 2016) For each subject the first 18 epochs of 16,384 samples (13.11 s) were used. The (pair-wise) corrected Amplitude Envelope Correlation (AECc) (Brookes et al., 2011, Bruns et al., 2000) was used as a measure of functional connectivity (FC) in the delta (0.5–4 Hz), theta (4–8 Hz), alpha1 (8–10 Hz), alpha2 (10–13 Hz), beta (13–30 Hz) and gamma (13–48 Hz) frequency bands (see Supplementary File 1). The AECc was computed per epoch for each pair of BNA regions, and then averaged over the epochs. Finally, AECc values were averaged globally (whole brain connectivity) and also averaged over regions in the oculomotor network.

### 2.4 Definition of saccades and the oculomotor network

Saccades are the fast eye movements that change our line of sight and can broadly be divided in reflexive and volitional saccades. Pro-saccades are made from a fixation point towards, and in response to, the appearance of a visual target, also called reflexive saccades. In contrast, the anti-saccade is one type of volitional saccade, (Leigh and Zee, 2015, Antoniades et al., 2013) evoked by generating a saccade in the direction away from the target, while suppressing reflexive pro-saccades and correctly estimating the mirror location. (Leigh and Zee, 2015, Nij Bijvank et al., 2018) Previous work has shown that especially anti-saccades involve widespread cortical processing, which has led to its use in cognitive, neurological and psychiatric research domains. (Leigh and Zee, 2015, Anderson and MacAskill, 2013) Fig. 1 provides a schematic overview and description of the oculomotor network involved in the control of eye movements. This network was defined using the FOCuS atlas, (Coiner et al., 2019) and divided in three main sub-regions (see Fig. 2 and Supplementary Table 1).

**Fig. 1:**
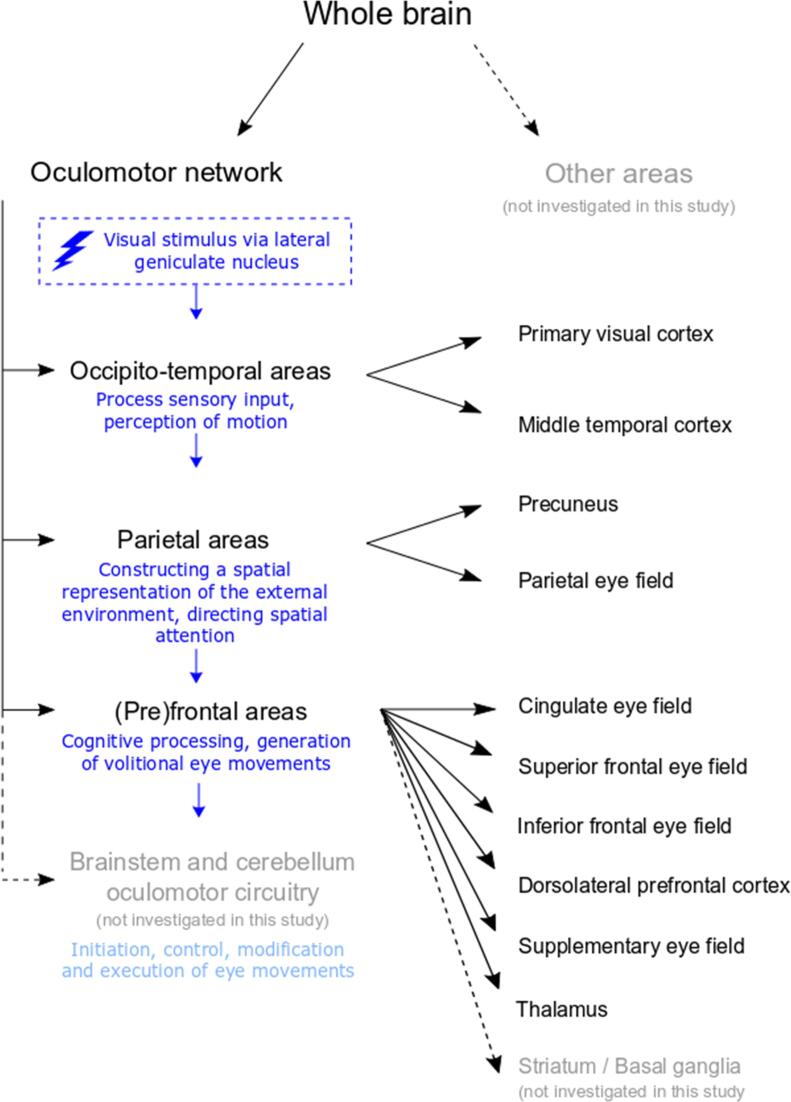
Schematic overview of the oculomotor network, in which the regions are highly interconnected to integrate sensory, motivational executive and motor information which are required for eye movement execution. Three main sub-regions (occipito-temporal, parietal and (pre)frontal) were defined and the corresponding subareas are indicated with the arrows. Visual stimuli travel from the retina, via the lateral geniculate nucleus, to the primary visual cortex located in the occipital cortex. Preliminary processing takes places in, amongst others, the middle temporal cortex, which is involved in the perception of motion. These regions are connected with the posterior parietal cortex (parietal eye field and precuneus), which are responsible for constructing a spatial representation of the environment and directing spatial attention. From the posterior parietal cortex, direct connections with the superior colliculus exist, which can generate reflexive eye movements. For more volitional eye movements and broad-sale cognitive processing, information passes from the parietal to the (pre)frontal cortex. Regions in this area are thought to be involved in, amongst others, decisional and predictive processes, performance monitoring and motivation and modulation of motor commands. The frontal regions are directly connected to the superior colliculus and indirectly via the basal ganglia, the latter connections are related to evaluating the significance of an action. A premotor circuit in the brainstem, including the superior colliculus, is responsible for initiation and direct control of eye movement. Cerebellar regions are crucial for fine-tuning of the eye movement, and project to the brainstem. The cerebellum, along with premotor regions in the brainstem, also projects to the thalamus. The thalamus is a widely connected region which is responsible for directing visual attention and relaying the various information from other sources necessary for oculomotor control.

**Fig. 2:**
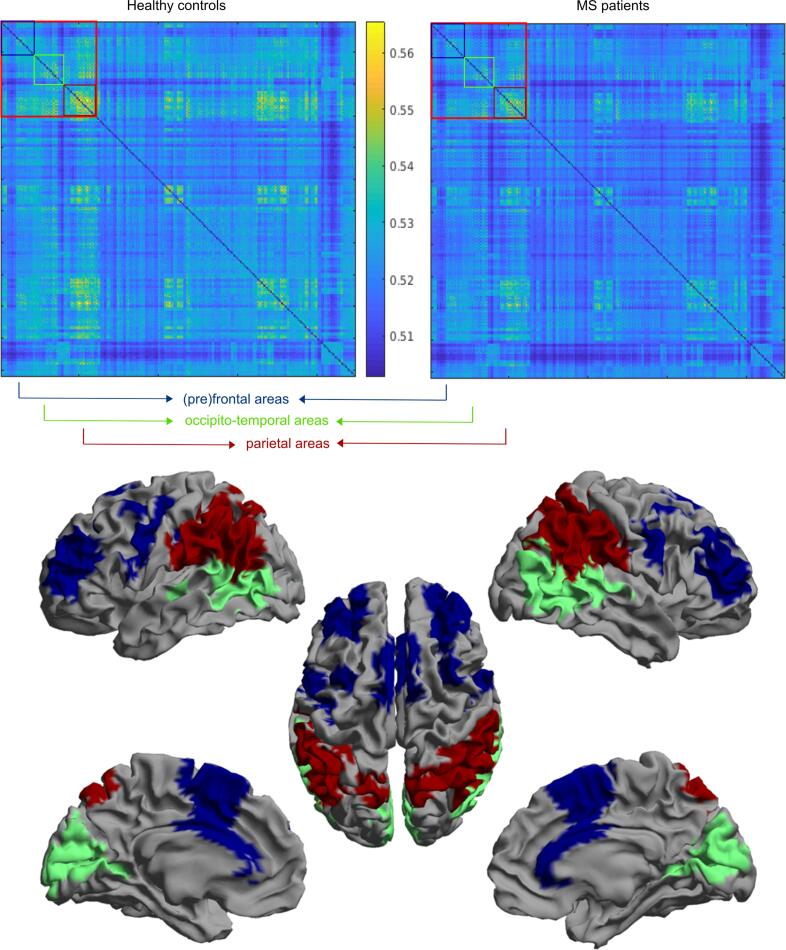
Oculomotor network definition. Top: functional connectivity matrix of the healthy control group (left) and the MS group (right). The colours in the matrices represent the values of the AECc between two ROIs, as indicated in the colour bar. The larger red square indicates the ROIs that are included in the oculomotor network. The smaller squares represent the three main sub-regions of the oculomotor network, that are indicated with the same colours in the brain plots (bottom): (pre)frontal (blue), occipito-temporal areas (green), and parietal areas (darker red). The thalamus is not shown in the brain plots, but is included in the (pre)frontal areas (see also Supplementary Table 1). (For interpretation of the references to colour in this figure legend, the reader is referred to the web version of this article.)

### 2.5 Neuropsychological evaluation

All subjects underwent an extensive neuropsychological evaluation as previously described, (Eijlers et al., 2017) using an extended Rao’s Brief Repeatable Battery of Neuropsychological tests (BRB-N) (Rao, 1990), including: Selective Reminding Test (verbal memory), Symbol Digit Modalities Test (information processing speed), Word List Generation Test (verbal fluency), 10/36 Spatial Recall Test (visuospatial memory), Concept Shifting Test (executive functioning), Stroop Colour Word test (attention), and Memory Comparison Test (working memory). Raw test scores were corrected for effects of age, sex, and level of education based on healthy controls using linear regression. Z-scores based on the distribution of healthy control scores were calculated for each individual domain and averaged across domains. (Eijlers et al., 2017, Amato et al., 2006)

### 2.6 Statistical analyses

Statistical analyses were performed using Stata (StataCorp. 2015. Stata Statistical Software: Release 14. College Station, TX: StataCorp LP) using independent t-tests (Gaussian data), Mann-Whitney U tests (non-Gaussian data) or chi-square tests (categorical data).

Next, associations between network and eye movement data were explored using linear regression models. A stepwise approach was used to evaluate if and to what extent narrowing down from global connectivity to regional connectivity within the oculomotor network regions was meaningful and to select the most relevant variables for our final multivariate model (i.e. a feasible number of variables for our sample size), in summary (for more details, see Supplementary File 2):

1. Global assessment, to identify associations between pro- and anti-saccadic performance and FC values in the different bands (whole brain, ocular motor network and its three main sub-regions). Variables showing significant relations were entered into step 2.
2. Regional associations, in which selected associations were further explored by zooming into individual areas within the oculomotor network
3. Multivariate regression, to evaluate the effect of adjustment for possible confounders. Next, we investigated which saccadic parameters were most strongly related to the specific FC value, by combining these parameters in one model. In the main text adjusted standardized beta’s are reported. Associations with a p-value lower than 0.01 were indicated in the result table (see Supplementary File 2 for more information).
4. Clinical relevance, as post-hoc analysis, we investigated if and which saccadic parameters resulting from step 3 were related to measures of clinical and cognitive functioning with linear and logistic regression models. Associations that survived the Holm-Bonferroni correction for multiple comparisons (Holm, 1979) were indicated in the result table. Furthermore, MS patients were divided in subgroups to evaluate the direction of results in comparison to healthy controls.

## 3 Results

In total 176 MS patients and 33 healthy controls were included in this study (see Table 1). Based on previous quality control (Nij Bijvank et al., 2019, Nij Bijvank et al., 2018) we excluded the pro-saccadic task of 8 subjects (7 MS, 1 control) and the anti-saccadic task of 4 MS patients. Neuropsychological data were available for 145 MS patients and 32 healthy controls.

**Table 1:** Demographic and clinical characteristics of the healthy controls and MS patients.

	**MS patients**	**Healthy controls**	**p-value**
	*N = 176*	*N = 33*	
Sex (N, female)	121 (69%)	21 (64%)	0.564
Age (years)	54.0 ± 10.8	48.5 ± 9.3	0.007
Level of education (median (range))^a^	5 (1–7)	6 (1–7)	0.059
Disease duration (years)	20.6 ± 8.4	N/A	N/A
EDSS score (median (IQR, range))	3.5 (2.5, 0.0–8.0)	N/A	N/A
Disease course			
Relapsing-remitting (N)	115 (65%)	N/A	N/A
Secondary progressive (N)	47 (27%)	N/A	N/A
Primary progressive (N)	11 (6%)	N/A	N/A
Unclassifiable	3 (2%)	N/A	N/A
Presence of internuclear ophthalmoplegia	58 (33%)	N/A	N/A
Average cognition (Z-score)	−1.07 ± 0.82	0.03 ± 0.57	<0.001

Patients had a mean disease duration of 20.6 (±8.4) years, a median EDSS score of 3.5 (IQR 2.5) with a relapsing-remitting disease course for 65%. MS patients showed a significant lower average cognition than healthy controls (Z-score of −1.07 ± 0.82 and 0.03 ± 0.57 respectively, *p* < .001. Altered saccadic performance has been published before, (Nij Bijvank et al., 2020) showing delayed latencies of both pro- and anti-saccades in MS compared to controls (latency of 15 degrees pro-saccades 207 ms and 225 ms respectively, *p* = .028), reduced gain of pro-saccades (0.95 and 0.92 respectively (non-INO only), *p* = .003) and an increased proportion of errors (0.50 and 0.37 respectively, *p* = .004). As previously published, abnormalities of latencies and proportion of errors were more pronounced in progressive MS patients compared to relapsing-remitting MS patients. (Nij Bijvank et al., 2020)

### 3.1 Step 1 and 2: Variables of interest

**Fig. 3:**
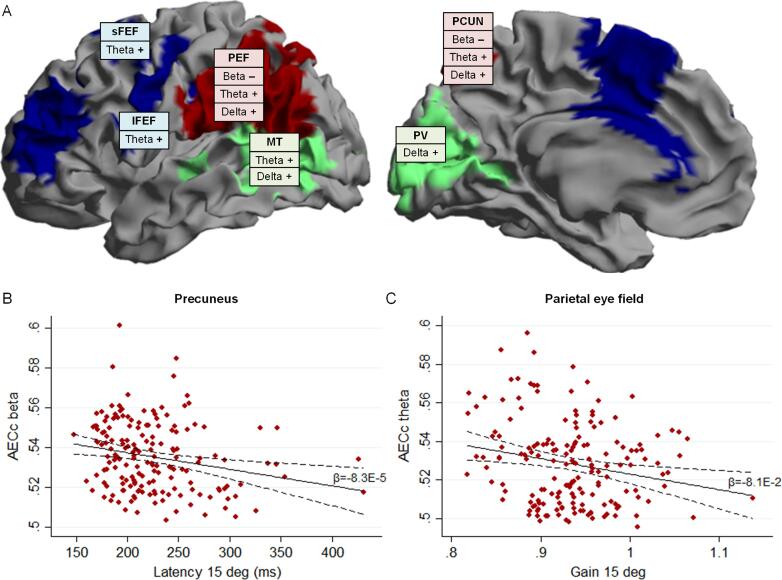
Associations between saccadic parameters and functional connectivity (FC) within areas of the oculomotor network in MS patients. A) Overview of the directions of the main results in the beta, theta and delta frequency bands. The boxes are indicating a positive (+) or negative (−) relation between FC and worse eye movement performance. Details of the associations are listed in Table 2. sFEF: superior frontal eye field; iFEF: inferior frontal eye field; PEF: parietal eye field; MT: middle temporal cortex; PCUN: precuneus; PV: primary visual cortex. B) and C) Exemplar scatterplots of associations between a saccadic parameter and FC (AECc) . The linear fit of the unadjusted association shown (solid line), with the corresponding unstandardized regression coefficient and 95% confidence interval (dashed lines). B) Association between latency of 15 degrees pro-saccades and beta band FC of the precuneus. C) Association between the gain of 15 degrees pro-saccades and theta band FC of the parietal eye field.

The screening step aimed to identify associations between saccadic parameters and FC as listed in Supplementary Table 2. The final associations and effects of adjustment for confounders are presented in Table 2, and the directions of the main results in Fig. 3A. For these associations, zooming in on the oculomotor network resulted in, on average, 13% higher effect sizes compared to whole brain associations. Moreover, zooming in further on main sub-regions resulted in, on average, 78% higher effect sizes compared to associations for the oculomotor network as a whole. For all but one of these associations, higher effect sizes were found for individual areas of the oculomotor network than for the larger region, with an average increase in effect size of 37%. Adjustment for confounders (age, sex and disease type) did not substantially change these effect sizes (Table 2). The results are summarized per type of saccadic parameter in the following section.

### 3.2 Step 3: Multivariate regression models

#### 3.2.1 Latencies

A higher latency of pro-saccades was associated with lower beta band FC for parietal and occipito-temporal areas, with the largest effect size for the precuneus (*β* = -0.21, *p* = .009, see Fig. 3B). In the anti-saccadic task (correct responses), higher latency associated with lower FC for the beta band in the precuneus (*β* = -0.17, *p* = .030). The latency of a correction of an incorrect response related to higher delta band FC, with the largest effect in the primary visual cortex (*β* = 0.19, *p* = .012). The latency of incorrect responses was not associated with FC.

#### 3.2.2 Errors

The proportion of errors in the anti-saccadic task was associated with higher FC (theta and delta band) of the parietal eye field, middle temporal cortex and the (pre)frontal sub-region (Table 2) and the largest effect was observed for theta band FC of the parietal eye field (*β* = 0.17, *p* = .028).

#### 3.2.3 Peak velocity

Lower peak velocity and Pv/Am were associated with higher theta band FC, as well as lower and upper alpha band FC (Table 2). The largest effect was found for theta band FC of the precuneus (adjusted model: *β* = -0.25, *p* = .007).

#### 3.2.4 Gain and final eye position

Lower gain (hypometric saccades) of pro-saccades associated with higher theta and delta band FC of distributed areas (Table 2). The largest effect was found for the association between the gain of 15 degrees pro-saccades and theta band FC within the parietal eye field (adjusted model: *β* = -0.24, *p* = .004), see Fig. 3C. Gain and error of the final eye position of anti-saccades were not related to FC.

#### 3.2.5 Sex effects

For latency of 8 degrees pro-saccades and proportion of errors in the anti-saccadic task, relevant effect modification by sex was found, explored further in Supplementary Table 3. In summary, latency of 8 degrees pro-saccades was negatively related to lower alpha band FC of parietal areas in female patients (adjusted model parietal eye field: *β* = -0.25, *p* = 0.010). In male patients higher gamma band FC of the inferior and superior frontal eye field and thalamus associated with a higher proportion of errors (adjusted model inferior frontal eye field: *β* = 0.29, *p* = 0.042).

#### 3.2.6 Combining saccadic parameters in one multivariate model

To assess which (combination of) eye movement parameter(s) related strongest to the functional network, FC values (specific frequency band – region combination) that were related to more than one saccadic parameter (Table 2) were investigated in an additional multivariate regression model. All relevant combined models involved theta band FC: Theta band FC of the middle temporal cortex was most strongly related to a combination of the proportion of errors in the anti-saccadic task (*β* = 0.18, *p* = .020) and the peak velocity of 15 degrees pro-saccades *(β* = -0.20, *p* = .026). Theta band FC for the parietal eye field was related to a combination of peak velocity (*β* = -0.23, *p* = .012) and gain (*β* = -0.22, *p* = .009) of 15 degrees. Similarly, theta band FC of the precuneus and inferior frontal eye field was related to a combination of peak velocity and gain of 15 degrees pro-saccades (precuneus: *β* = -0.22, *p* = .014 and *β* = -0.19, *p* = .025, respectively; inferior frontal eye field: *β* = -0.21, *p* = .021 and *β* = -0.20, *p* = .015, respectively).

**Table 2:** Results of multivariate regression analyses of the strongest associations between eye movement parameters and functional connectivity.

**Eye movement parameter**	**FC frequency band**	**Region / Area**	**Model 1**		**Model 2**		**Model 3**			
			B	p	B	p	B	95% CI	β	p
Latency 15 PS	Beta	Precuneus	−8.3	**0.003**	−8.7	**0.004**	−8.4	−14.5–−2.4	−0.23	**0.006**
		Parietal eye field	−7.6	**0.003**	−7.9	**0.004**	−6.9	−12.3–−1.5	−0.20	0.013
		Middle temporal	−4.4	0.046	−6.2	**0.008**	−5.4	−10.4–−0.7	−0.19	0.025
Latency 8 PS	Beta	Precuneus	−10.1	**0.005**	−10.1	**0.007**	−10.0	−17.5–−2.6	−0.21	**0.009**
		Parietal eye field	−7.9	0.015	−7.8	0.021	−6.8	−13.5–−0.1	−0.16	0.048
Latency correct response AS	Beta	Precuneus	−4.5	0.018	−4.5	0.019	−4.2	−8.1–−0.4	−0.17	0.030
	Delta	Primary visual	2.6	0.033	2.5	0.038	2.2	−0.3–4.7	0.14	0.084
		Middle temporal	2.7	0.039	2.7	0.038	2.5	−0.2–5.1	0.15	0.067
Latency correction AS	Delta	Primary visual	2.2	**0.005**	2.1	**0.005**	1.9	0.4–3.5	0.19	0.012
		Parietal eye field	1.4	0.023	1.4	0.019	1.4	1.1–2.6	0.15	0.033
Proportion errors AS	Theta	Middle temporal	14.3	0.015	14.2	0.017	14.9	2.9–26.7	0.19	0.015
		Parietal eye field	14.1	0.038	14.9	0.029	15.5	1.7–29.3	0.17	0.028
		(Pre)frontal sub-region	8.9	0.026	9.2	0.021	8.7	0.7–16.7	0.17	0.033
	Delta	Middle temporal	8.3	0.024	8.3	0.025	7.1	−0.4–14.6	0.15	0.062
Peak velocity 15 PS	Theta	Precuneus	−7.4	**0.006**	−7.6	**0.006**	−7.5	−12.9–−2.1	−0.25	**0.007**
		Parietal eye field	−6.8	**0.006**	−7.0	**0.005**	−7.1	−12.1–−2.1	−0.25	**0.005**
		Inferior frontal eye field	−5.4	**0.009**	−5.5	**0.007**	−5.4	−9.4–−1.3	−0.23	0.010
		Middle temporal	−5.4	0.013	−5.5	0.011	−5.5	−9.8–−1.2	−0.23	0.013
	Lower alpha	Supplementary eye field	−5.8	**0.005**	−5.8	**0.005**	−5.5	−9.6–−1.3	−0.23	0.010
Peak velocity 8 PS	Theta	Precuneus	−7.1	0.026	−7.1	0.026	−7.1	−13.4–−0.8	−0.20	0.029
		Parietal eye field	−6.7	0.023	−6.7	0.021	−6.8	−12.6–−1.0	−0.21	0.023
		Middle temporal	−5.9	0.020	−5.9	0.019	−6.0	−11.0–−1.0	−0.21	0.019
Pv/Am 15 PS	Upper alpha	Precuneus	−8.6	0.030	−8.7	0.030	−8.8	−16.7–−0.9	−0.20	0.030
Pv/Am 8 PS	Theta	Middle temporal	−5.0	0.031	−5.0	0.030	−5.0	−9.6–−0.5	−0.21	0.031
Gain 15 PS	Theta	Parietal eye field	−87.3	**0.005**	−92.2	**0.003**	−93.1	−155.7–−30.0	−0.24	**0.004**
		Precuneus	−77.9	0.024	−82.6	0.017	−88.0	−156.5–−19.5	−0.21	0.012
		Inferior frontal eye field	−68.0	**0.008**	−70.7	**0.006**	−70.7	−122.0–−19.5	−0.22	**0.007**
	Delta	Precuneus	−35.9	**0.008**	−37.3	**0.006**	−40.7	−67.6–−13.9	−0.25	**0.003**
Gain 8 PS	Theta	Parietal eye field	−77.2	**0.006**	−75.4	**0.007**	−74.2	−129.8–−18.5	−0.21	**0.009**
		Precuneus	−65.8	0.032	−63.3	0.040	−59.6	−120.6–−13.8	−0.15	0.055
		Inferior frontal eye field	−71.9	**0.002**	−71.8	**0.002**	−68.1	−113.1––23.0	−0.23	**0.003**
		Superior frontal eye field	−55.9	0.017	−53.2	0.021	−54.8	−99.6–−9.9	−0.18	0.017
		Middle temporal	−52.8	0.029	−52.8	0.031	−48.3	−96.5–0.0	−0.15	0.050
	Delta	Precuneus	−28.3	0.019	−27.9	0.022	−28.9	−52.8–−5.0	−0.19	0.018
		Middle temporal	−31.7	0.033	–32.8	0.028	−31.9	−61.3–−2.4	−0.17	0.034

Bold p-values (p) represents values lower than 0.01. Unstandardized regression coefficients (B) are all multiplied by a factor of 10,000 and are presented per 10 ms for latency, per 10 degrees/second for peak velocity, per 1 degree/second/degree for Pv/Am per 1 degree for the error of the final eye position and per 0.1 for gain and proportion of errors. Model 1: raw association; Model 2: association adjusted for age and sex; Model 3: association adjusted for age, sex and disease type. In model 3 the 95% confidence interval (CI) of B and the standardized regression coefficient (β) are additionally listed. In all models, parameters that are directly influenced by internuclear ophthalmoplegia (peak velocity, Pv/Am and gain) are additionally adjusted for the presence of unilateral or bilateral internuclear ophthalmoplegia. FC: functional connectivity; PS: pro-saccades; AS: anti-saccades; 15: saccades made in response to target amplitude of 15 degrees of visual angle; 8: saccades made in response to target amplitude of 8 degrees of visual angle; Pv/Am: peak velocity divided by amplitude; CI: confidence interval.

### 3.3 Step 4: Disability and cognition

**Fig. 4:**
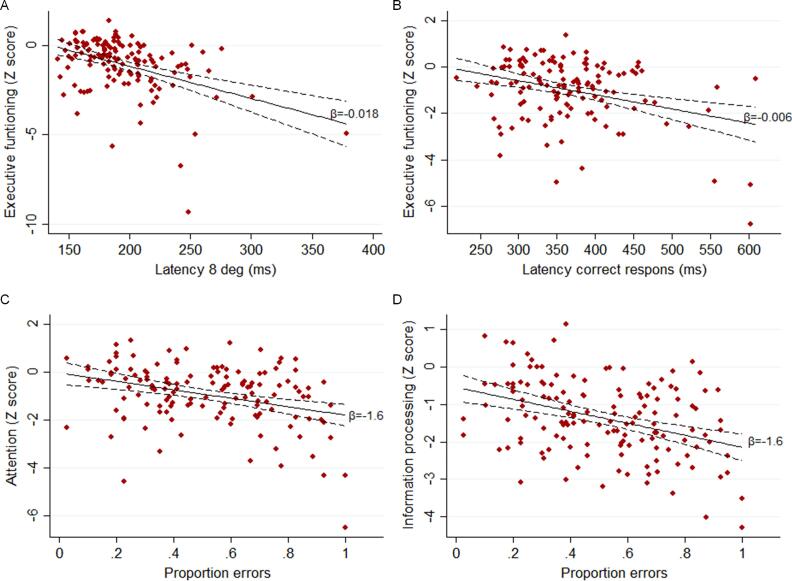
Exemplar scatterplots of associations between saccadic parameters and cognitive domain Z-scores of MS patients. The linear fit of the unadjusted association is shown, with the corresponding unstandardized regression coefficients and 95% confidence interval (dashed lines). A) Association between latency of 8 degrees pro-saccades and the executive functioning Z-score. B) Association between the latency of correct responses in the anti-saccadic task and the attention Z-score. C) Association between the proportion of errors in the anti-saccadic task and the attention Z-score. D) Association between the proportion of errors in the anti-saccadic task and the information processing Z-score.

Relations between clinical measures and saccadic parameters are shown in Table 3. In summary, in MS, a higher latency (of pro-saccades and correction in the anti-saccadic task) related to longer disease durations and higher EDSS. Furthermore, higher latency of 15 degrees saccades related to worse executive functioning (*β* = -0.49, *p* < .001), information processing speed (*β* = -0.37, *p* < .001) and working memory (*β* = -0.36, *p* < .001). Latency of 8 degrees saccades and latency of correct anti-saccades showed similar relations. Higher latency of a correction in the anti-saccadic task related to worse attention (*β* = -0.22, *p* = .015). More errors associated with a lower score in five domains, most strongly information processing speed (*β* = -0.36, *p* < .001) and attention (*β* = -0.31, *p* = .001). Peak velocity, Pv/Am and gain of pro-saccades were not strongly related to clinical and cognitive function. Fig. 4 shows examples of abovementioned relations.

**Table 3:** Results of multivariate regression analyses of associations between eye movement parameters and both clinical variables and cognitive domain Z-scores.

**Eye movement parameter**	**Clinical variable / Cognitive domain**	**Adjusted model**			
		OR / B	95% CI	β	p-value
Latency 15 degrees PS	Disease duration	1.12	1.04–1.21	N/A	**0.003**
	EDSS	1.17	1.07–1.27	N/A	**0.001**
	Executive functioning	−0.16	−0.22–−0.11	−0.49	**<0.001**
	Information processing	−0.08	−0.11–−0.04	−0.37	**<0.001**
	Visuospatial memory	−0.06	−0.11–−0.01	−0.21	0.027
	Working memory	−0.10	−0.15–−0.05	−0.36	**<0.001**
Latency 8 degrees PS	Disease duration	1.16	1.06–1.29	N/A	**0.003**
	EDSS	1.24	1.10–1.39	N/A	**<0.001**
	Executive functioning	−0.19	−0.26–−0.12	−0.44	**<0.001**
	Information processing	−0.06	−0.11–−0.02	−0.25	**0.005**
	Working memory	−0.12	−0.18–−0.06	−0.34	**<0.001**
Latency correct response AS	Disease course	1.05	1.00–1.11	N/A	0.044
	EDSS	1.07	1.01–1.12	N/A	**0.011**
	Executive functioning	−0.06	−0.09–−0.03	−0.32	**<0.001**
	Information processing	−0.04	−0.06–−0.02	−0.29	**0.001**
	Working memory	−0.04	−0.08–−0.01	−0.24	**0.008**
	Attention	−0.04	−0.07–−0.01	−0.23	0.012
Latency correction AS	Executive functioning	−0.05	−0.07–−0.03	−0.37	**<0.001**
	Information processing	−0.03	−0.05–−0.01	−0.32	**<0.001**
	Working memory	−0.04	−0.06–−0.02	−0.32	**<0.001**
	Attention	−0.02	−0.04–−0.00	−0.22	**0.015**
Proportion errors AS	Executive functioning	−0.16	−0.27–−0.04	−0.25	**0.007**
	Verbal memory	−0.13	−0.21–−0.04	−0.25	**0.003**
	Information processing	−0.16	−0.23–−0.09	−0.36	**<0.001**
	Verbal fluency	−0.08	−0.15–−0.02	−0.21	**0.016**
	Attention	−0.16	−0.25–−0.07	−0.31	**0.001**
Gain 8 degrees PS	Disease duration		1.23–3.41	N/A	**0.010**
	Verbal fluency	0.25	0.19–0.49	0.18	0.034

Bold p-values represent p-values of associations that survived multiple comparisons correction (which was performed for clinical variables and cognitive domains separately). Odds ratios (OR) and unstandardized regression coefficients (B) are presented per 10 ms for latencies, per 10 degrees/second for peak velocities, per 1 degree/second/degree for Pv/Am and per 0.1 for gain and proportion of errors. Logistic regression analyses were used for the associations with clinical variables: disease duration (median split, <21 versus ≥ 21 years), EDSS (<4.5 versus ≥ 4.5) and disease course (relapsing-remitting versus secondary and primary progressive), adjusted for age and sex ((the relation with disease duration only for sex due to collinearity with age). The associations with cognitive domain Z-scores were investigated with linear regression analyses, adjusted for age, sex, level of education and disease type. The associations of gain, peak velocity and Pv/Am are additionally adjusted for the presence of unilateral or bilateral internuclear ophthalmoplegia. PS: pro-saccades; AS: anti-saccades; 15 degrees: saccades made in response to target amplitude of 15 degrees of visual angle; 8 degrees: saccades made in response to target amplitude of 8 degrees of visual angle; CI: confidence interval; β: standardized regression coefficient.

#### 3.3.1 Subgroup analysis

**Fig. 5:**
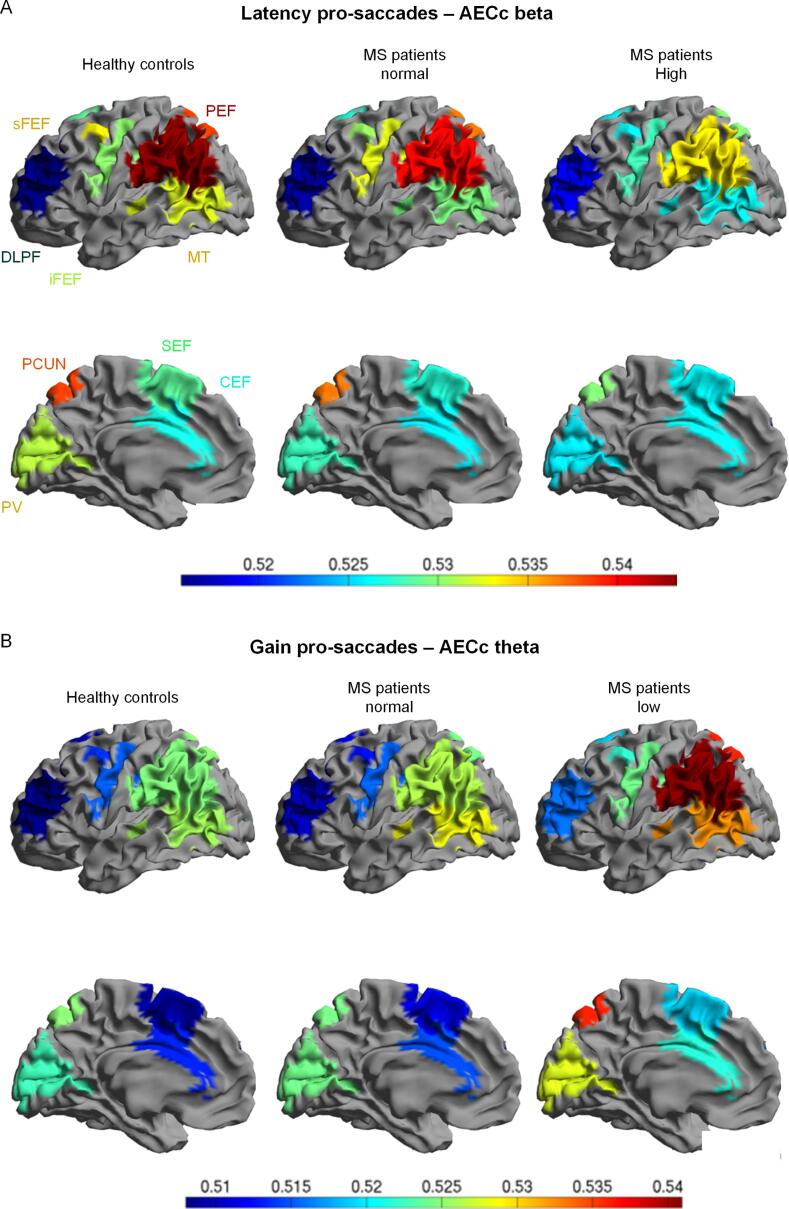
Brain plots showing functional connectivity (AECc values as indicated in the colour scale bars) of different areas of the oculomotor network, dichotomized in healthy controls (left), less impaired on eye movements (middle) and more impaired on eye movements (right) MS patients. A) Latency of pro-saccades and FC of the beta band. B) Gain of pro-saccades and FC of the theta band. CEF: cingulate eye field; DLPF: dorsolateral prefrontal cortex; iFEF: inferior frontal eye field; MT: middle temporal cortex; PEF: parietal eye field; PCUN: precuneus; PV: primary visual cortex; SEF: supplementary eye field; sFEF: superior frontal eye field.

Finally, subgroups were formed using controls as a reference, to study effects in patients with saccadic parameters that were abnormal (Table 2). This was only done for variables showing the strongest associations with FC, i.e. for latencies of pro-saccades (beta band) and gain of pro-saccades (theta/delta bands). The MS patients were therefore divided in 1) a normal and a high latency subgroup, 2) a normal and a low gain subgroup. In Fig. 5, FC values (beta and theta band) for MS subgroups and healthy controls are visualized for areas of the oculomotor network. Patients who displayed high latency and low gain generally showed a larger FC deviation from controls (i.e. lower beta band FC, higher theta band FC), than did the patients with normal saccadic parameters. The same pattern was observed for low gain and higher delta band FC. Taken into account the regions of Table 2, the FC was significantly different between the abnormal saccadic group and healthy control group for the parietal eye field (latency/beta band and gain /theta band), inferior frontal eye field (gain/theta band) and the middle temporal cortex (gain/delta band).

## 4 Discussion

This study showed that altered saccadic parameters in MS are related to FC of the oculomotor network. Regionally, latency was most strongly related to FC of parietal areas, errors, gain and peak velocity additionally to frontal FC. Saccadic parameters were also related to clinical outcomes, especially cognitive function.

Our study identified that abnormal eye movements in MS are mostly related to higher delta and theta, but lower beta FC, which was not studied before. Most previous studies either used other modalities, other measures of network function (power, coherence or different FC measures), or investigate task-related responses, which makes direct comparison of our results not feasible. Signals in the delta band are often attributed to artefacts, typically in relation to blinks and eye movement (Bodala et al., 2016), or are linked to sleep and deep levels of relaxation. (Mandal et al., 2018) However, increased delta and theta band power and FC can also reflect pathologic conditions, amongst others shown in malignancies of the brain and MS. (DEJONGH et al., 2003, Tewarie et al., 2014) Beta waves are dominant in normal conscious states and during attentive cognitive tasks, and theta band rhythms are associated with drowsiness or sleep, as well as memory and learning. (Mandal et al., 2018) In general, the pattern of lower FC in the beta band and higher FC in theta/delta band could indicate generalized slowing of the network, which can be considered as an indicator of neurodegenerative processes, such as in Lewy Bodies dementia, but has also been described in MS. (Tewarie et al., 2014, Dauwan et al., 2018) This generalized slowing has also been related to cognitive dysfunction in MS, (Schoonhoven et al., 2018) to which our results also add relevance for eye movement abnormalities.

Regarding the specific localization of abnormalities within the network, our results showed that a higher latency of pro-saccades (a longer reaction time of reflexive movements) related to a lower beta band FC of the precuneus and parietal eye field (PEF). The PEF is located in the posterior intraparietal sulcus (IPS). Previous work has shown that the PEF plays a role in attention and visuospatial integration in conjunction with other regions in the IPS and is crucial for generation of reflexive saccades. (Coiner et al., 2019) The precuneus has also been implicated in eye movement control. (Leigh and Zee, 2015, Coiner et al., 2019) Although this region is not specific for oculomotor function, it has an important role in overall network functioning as part of the default-mode network, as well as for attention and visuospatial processing. (Coiner et al., 2019) A higher latency of pro-saccades can potentially result from a delay anywhere in the afferent or efferent visual system, for which we now shown involvement of these two parietal areas in MS.

This study supports the hypothesis that abnormal saccadic parameters can reflect clinically relevant dysfunction in the functional brain network. Especially, our results confirmed that an altered latency of saccades might reflect a broad range of cognitive functions, including processing of visual information, task planning, attention and selection of relevant stimuli. (Leigh and Zee, 2015) This might indicate that latency partly reflects the reaction time / speed component that contributes to the performance on different cognitive tests as well. This could indicate involvement of the same underpinning network, which should be studied further. Peak velocity and gain of pro-saccades showed strong relations with theta band FC in the regression models, but were less consistently related to clinical variables and cognitive function. This suggests that these parameters reflect network dysfunction that is not directly related to other (clinical) outcomes.

The anti-saccadic task of our study represents volitional eye movements and theoretically requires more broad scale cognitive processing and involvement of frontal regions. Although associations of anti-saccadic parameters with FC were weaker than for pro-saccadic parameters, our results do suggest that the proportion of errors in this task was related to a more widespread and less region-specific increase in theta band FC of MS patients. We did not find a significant relation specifically with FC of the dorsolateral prefrontal cortex though, a region traditionally associated with the number of errors in the anti-saccadic task. (Leigh and Zee, 2015) The proportion of errors did relate strongly to cognitive function in multiple domains. The relation with network dysfunction was potentially not fully captured by the connectivity measure chosen in this study, which is a conservative measure that avoids spurious connections that are due to the effects of volume conduction/field spread at the cost of ignoring true zero-lag functional interactions.

We also observed sex effects (Supplementary Table 3), which might even have been underestimated due to the stepwise approach in the analyses; we only tested effect modification in the final model. Nevertheless, our findings underline that sex differences are important to take into account in MS studies, especially in network studies, because there is evidence that both the alterations to functional wiring of the brain and the severity of MS pathology are especially severe in the male MS brain. (Schoonheim et al., 2012)

Some potential limitations of our study have to be considered. MEG generally has a lower spatial resolution than functional MRI (fMRI), although its spatial resolution has recently improved in newer models. (Hillebrand et al., 2012, Gross, 2019) It is also a less commonly used modality in MS, which makes it more difficult to compare our results with previous studies. Although recent studies have demonstrated that the sensor-space data of MEG can be accurately projected to deeper brain regions, (Hillebrand et al., 2016) whether the same also holds for the brainstem or cerebellum remains unclear. These structures are relevant for accurate eye movements, therefore associations with pathology in these structures would be relevant to study in more detail, e.g. relations between FC, lesion volume and measures of atrophy The higher temporal resolution of MEG is an advantage compared to fMRI, as it allows for extension of our approach to more sophisticated parameters, such as high-resolution dynamic FC, (Tewarie et al., 2019) in future studies. Another potential limitation is the definition of the oculomotor network. Exact locations of areas involved in eye movement control have not always been clearly delineated, (Leigh and Zee, 2015) can vary between individuals, or are not accurately represented in different atlases. Nevertheless, we did find associations for the defined areas, most frequently stronger than for the whole brain or the oculomotor network as a whole. However, we did not investigate specific regions outside this oculomotor network. A next step could be to use more advanced statistical approaches, for example deep-learning algorithms, which could potentially select the most relevant regions and interactions between regions that are involved in oculomotor dysfunction, in a non-hypothesis driven manner. Another point to consider is that we included MS patients with a relatively long disease duration and as part of an on-going cohort. We cannot draw conclusions on the occurrence of eye movement abnormalities and the relation to network dysfunction in patients during early stages of the disease. A methodological challenge for the study was the number of potential statistical comparisons, which were reduced by our stepwise approach in the statistical analysis. This was specifically used to show that zooming in towards regional measures yields stronger clinical relations, which should be validated in another patient sample. Finally, the cross-sectional design of our study limits the ability to draw conclusions about the order in which brain and eye movement abnormalities occur, and their causal relationships. Mediation analyses in a longitudinal dataset could help to crystallize the expected causal chain of (1) structural damage, (2) brain network dysfunction and (3) eye movement abnormalities (as well as other clinical outcomes).

To conclude, this study indicates that eye movement disorders are related to functional network changes of the oculomotor network and also strongly relate to cognitive impairment in MS. Latency, gain and peak velocity of pro-saccades were most strongly related to FC and regional oculomotor network changes were more relevant than global network changes in this MEG study. To further elucidate the relation with network dysfunction, future work should focus on more complex network measures, longitudinal associations and also include FC of the brainstem and cerebellum.

## Funding

This work was supported by the Dutch MS Research Foundation, grant nr. 18-1006MS.

## CRediT authorship contribution statement

**J.A. Nij Bijvank:** Conceptualization, Methodology, Software, Validation, Formal analysis, Investigation, Data curation, Writing – original draft, Visualization, Project administration, Funding acquisition. **E.M.M. Strijbis:** Conceptualization, Methodology, Validation, Data curation, Writing – review & editing, Supervision, Funding acquisition. **I.M. Nauta:** Methodology, Software, Validation, Formal analysis, Data curation, Writing – review & editing. **S.D. Kulik:** Methodology, Software, Validation, Formal analysis, Data curation, Writing – review & editing. **L.J. Balk:** Formal analysis, Writing – review & editing, Supervision. **C.J. Stam:** Methodology, Software, Validation, Resources, Data curation, Writing – review & editing. **A. Hillebrand:** Methodology, Software, Validation, Resources, Data curation, Writing – review & editing. **J.J.G. Geurts:** Resources, Writing – review & editing, Supervision. **B.M.J. Uitdehaag:** Resources, Writing – review & editing, Funding acquisition, Supervision. **L.J. Rijn:** Conceptualization, Methodology, Validation, Resources, Writing – review & editing, Supervision, Funding acquisition. **A. Petzold:** Conceptualization, Methodology, Validation, Resources, Writing – review & editing, Supervision, Funding acquisition. **M.M. Schoonheim:** Conceptualization, Methodology, Software, Validation, Resources, Data curation, Writing – review & editing, Supervision, Funding acquisition.

## Declaration of Competing Interest

J.A. Nij Bijvank is supported by the Dutch MS Research Foundation, grant nr. 18-1027. Serves on the editorial board (literature review) of Neuro-Ophthalmology. E.M. Strijbis reports no disclosures. I.M. Nauta is supported by the Dutch MS Research Foundation, grant nr. 15-911 -S. Kulik reports no disclosures. L.J. Balk reports no disclosures. C.J. Stam reports no disclosures. A. Hillebrand serves on the editorial board of Scientific Reports. J.J.G. Geurts is an editor of MS journal and serves on the editorial boards of Neurology and Frontiers of Neurology and is president of the Netherlands organization for health research and innovation andhas served as a consultant for Merck-Serono, Biogen, Novartis, Genzyme and Teva Pharmaceuticals. B.M.J. Uitdehaag has received consultancy fees from Biogen Idec, Genzyme, Merck Serono, Novartis, Roche and Teva. L.J. van Rijn reports no disclosures. A. Petzold reports personal fees from Novartis, Heidelberg Engineering, Zeiss, grants from Novartis,outside the submitted work; and is part of the steering committee of the OCTiMS study which issponsored by Novartis and the Angio-OCT steering committee which is sponsored by Zeiss. He doesnot receive compensation for these activities -M.M. Schoonheim serves on the editorial board of Frontiers of Neurology, receives research supportfrom the Dutch MS Research Foundation, and has received compensation for consulting services orspeaker honoraria from ExceMed, Genzyme and Biogen.

## References

[b0005] Mollison D., Sellar R., Bastin M., Mollison D., Chandran S., Wardlaw J., Connick P., Aktas O. (2017). The clinico-radiological paradox of cognitive function and MRI burden of white matter lesions in people with multiple sclerosis: A systematic review and meta-analysis. PLoS ONE.

[b0010] Schoonheim M.M., Geurts J.J.G., Wiebenga O.T., De Munck J.C., Polman C.H., Stam C.J., Barkhof F., Wink A.M. (2014). Changes in functional network centrality underlie cognitive dysfunction and physical disability in multiple sclerosis. Mult Scler.

[b0015] Schoonheim M.M., Meijer K.A., Geurts J.J. (2015). Network collapse and cognitive impairment in multiple sclerosis. Front. Neurol..

[b0020] Nauta IM, Kulik SD, Breedt LC, et al. Functional brain network organization measured with magnetoencephalography predicts cognitive decline in multiple sclerosis. Mult. Scler. 2020:1352458520977160. doi: 10.1177/1352458520977160 [published Online First: 2020/12/10]. 10.1177/1352458520977160 PMC8474326 33295249

[b0025] Tewarie P., Schoonheim M.M., Schouten D.I., Polman C.H., Balk L.J., Uitdehaag B.M.J., Geurts J.J.G., Hillebrand A., Barkhof F., Stam C.J. (2015). Functional brain networks: linking thalamic atrophy to clinical disability in multiple sclerosis, a multimodal fMRI and MEG study. Hum. Brain Mapp..

[b0030] Sjøgård M., Wens V., Van Schependom J., Costers L., D'hooghe M., D'haeseleer M., Woolrich M., Goldman S., Nagels G., De Tiège X. (2021). Brain dysconnectivity relates to disability and cognitive impairment in multiple sclerosis. Hum. Brain Mapp..

[b0035] van Munster C.E., Uitdehaag B.M. (2017). Outcome Measures in Clinical Trials for Multiple Sclerosis. CNS Drugs.

[b0040] Leigh RJ, Zee DS. 2015. The neurology of eye movements. 5 ed. Oxford: Oxford University Press.

[b0045] Nij Bijvank J.A., van Rijn L.J., Balk L.J., Tan H.S., Uitdehaag B.M.J., Petzold A. (2019). Diagnosing and quantifying a common deficit in multiple sclerosis: internuclear ophthalmoplegia. Neurology.

[b0050] Jasse L., Vukusic S., Durand-Dubief F., Vartin C., Piras C., Bernard M., Pélisson D., Confavreux C., Vighetto A., Tilikete C. (2013). Persistent visual impairment in multiple sclerosis: prevalence, mechanisms and resulting disability. Mult Scler.

[b0055] Nij Bijvank J.A., Petzold A., Coric D., Tan H.S., Uitdehaag B.M.J., Balk L.J., van Rijn L.J. (2020). Saccadic delay in multiple sclerosis: A quantitative description. Vision Res..

[b0060] Fielding J., Clough M., Beh S., Millist L., Sears D., Frohman A.N., Lizak N., Lim J., Kolbe S., Rennaker R.L., Frohman T.C., White O.B., Frohman E.M. (2015). Ocular motor signatures of cognitive dysfunction in multiple sclerosis. Nat. Rev. Neurol..

[b0065] Kincses B., Hérák B.J., Szabó N., Bozsik B., Faragó P., Király A., Veréb D., Tóth E., Kocsis K., Bencsik K., Vécsei L., Kincses Z.T. (2019). Gray Matter Atrophy to Explain Subclinical Oculomotor Deficit in Multiple Sclerosis. Front. Neurol..

[b0070] Nij Bijvank J.A., Petzold A., Balk L.J., Tan H.S., Uitdehaag B.M.J., Theodorou M., van Rijn L.J., Anderson A. (2018). A standardized protocol for quantification of saccadic eye movements: DEMoNS. PLoS ONE.

[b0075] Frohman T.C., Frohman E.M., O'Suilleabhain P., Salter A., Dewey R.B., Hogan N., Galetta S., Lee A.G., Straumann D., Noseworthy J., Zee D., Corbett J., Corboy J., Rivera V.M., Kramer P.D. (2003). Accuracy of clinical detection of INO in MS: Corroboration with quantitative infrared oculography. Neurology.

[b0080] Sheehy C.K., Bensinger E.S., Romeo A., Rani L., Stepien-Bernabe N., Shi B., Helft Z., Putnam N., Cordano C., Gelfand J.M., Bove R., Stevenson S.B., Green A.J. (2020). Fixational microsaccades: A quantitative and objective measure of disability in multiple sclerosis. Mult. Scler..

[b0085] Nij Bijvank J.A., Petzold A., Coric D., Tan H.S., Uitdehaag B.M.J., Balk L.J., van Rijn L.J. (2019). Quantification of Visual Fixation in Multiple Sclerosis. Invest. Ophthalmol. Vis. Sci..

[b0090] Coiner B., Pan H., Bennett M.L., Bodien Y.G., Iyer S., O’Neil-Pirozzi T.M., Leung L., Giacino J.T., Stern E. (2019). Functional neuroanatomy of the human eye movement network: a review and atlas. Brain Struct. Funct..

[b0095] Polman C.H., Reingold S.C., Banwell B., Clanet M., Cohen J.A., Filippi M., Fujihara K., Havrdova E., Hutchinson M., Kappos L., Lublin F.D., Montalban X., O'Connor P., Sandberg‐Wollheim M., Thompson A.J., Waubant E., Weinshenker B., Wolinsky J.S. (2011). Diagnostic criteria for multiple sclerosis: 2010 revisions to the McDonald criteria. Ann. Neurol..

[b0100] Lublin F.D., Reingold S.C. (1996). Defining the clinical course of multiple sclerosis: Results of an international survey. National Multiple Sclerosis Society (USA) Advisory Committee on Clinical Trials of New Agents in Multiple Sclerosis. Neurology.

[b0105] Kurtzke J.F. (1983). Rating neurological impairment in multiple sclerosis: An expanded disability status scale (EDSS). Neurology.

[b0110] Antoniades C., Ettinger U., Gaymard B., Gilchrist I., Kristjánsson A., Kennard C., John Leigh R., Noorani I., Pouget P., Smyrnis N., Tarnowski A., Zee D.S., Carpenter R.H.S. (2013). An internationally standardised antisaccade protocol. Vision Res..

[b0115] Hillebrand A., Tewarie P., van Dellen E., Yu M., Carbo E.W.S., Douw L., Gouw A.A., van Straaten E.C.W., Stam C.J. (2016). Direction of information flow in large-scale resting-state networks is frequency-dependent. Proc. Natl. Acad. Sci. U.S.A..

[b0120] Taulu S., Simola J. (2006). Spatiotemporal signal space separation method for rejecting nearby interference in MEG measurements. Phys. Med. Biol..

[b0125] Fan L., Li H., Zhuo J., Zhang Y.u., Wang J., Chen L., Yang Z., Chu C., Xie S., Laird A.R., Fox P.T., Eickhoff S.B., Yu C., Jiang T. (2016). The Human Brainnetome Atlas: A New Brain Atlas Based on Connectional Architecture. Cereb. Cortex.

[b0130] Hillebrand A., Barnes G.R., Bosboom J.L., Berendse H.W., Stam C.J. (2012). Frequency-dependent functional connectivity within resting-state networks: an atlas-based MEG beamformer solution. Neuroimage.

[b0135] Brookes M.J., Hale J.R., Zumer J.M., Stevenson C.M., Francis S.T., Barnes G.R., Owen J.P., Morris P.G., Nagarajan S.S. (2011). Measuring functional connectivity using MEG: methodology and comparison with fcMRI. Neuroimage.

[b0140] Bruns A, Eckhorn R, Jokeit H, et al. Amplitude envelope correlation detects coupling among incoherent brain signals. Neuroreport 2000;11(7):1509-14 [published Online First: 2000/06/07]. 10841367

[b0145] Anderson T.J., MacAskill M.R. (2013). Eye movements in patients with neurodegenerative disorders. Nat. Rev. Neurol..

[b0150] Eijlers A.J.C., Meijer K.A., Wassenaar T.M., Steenwijk M.D., Uitdehaag B.M.J., Barkhof F., Wink A.M., Geurts J.J.G., Schoonheim M.M. (2017). Increased default-mode network centrality in cognitively impaired multiple sclerosis patients. Neurology.

[b0155] Rao S.M. (1990).

[b0160] Amato M.P., Portaccio E., Goretti B., Zipoli V., Ricchiuti L., De Caro M.F., Patti F., Vecchio R., Sorbi S., Trojano M. (2006). The Rao's Brief Repeatable Battery and Stroop Test: normative values with age, education and gender corrections in an Italian population. Mult. Scler..

[b0165] Holm S. (1979). A simple sequentially rejective multiple test procedure. Scand. J. Statist..

[b0170] Bodala I.P., Li J., Thakor N.V. (2016). EEG and Eye Tracking Demonstrate Vigilance Enhancement with Challenge Integration. Front. Hum. Neurosci..

[b0175] Mandal P.K., Banerjee A., Tripathi M. (2018). A Comprehensive Review of Magnetoencephalography (MEG) Studies for Brain Functionality in Healthy Aging and Alzheimer's Disease (AD). Front. Comput. Neurosci..

[b0180] DEJONGH A., BAAYEN J., DEMUNCK J., HEETHAAR R., VANDERTOP W., STAM C. (2003). The influence of brain tumor treatment on pathological delta activity in MEG. Neuroimage.

[b0185] Tewarie P., Steenwijk M.D., Tijms B.M., Daams M., Balk L.J., Stam C.J., Uitdehaag B.M.J., Polman C.H., Geurts J.J.G., Barkhof F., Pouwels P.J.W., Vrenken H., Hillebrand A. (2014). Disruption of structural and functional networks in long-standing multiple sclerosis. Hum. Brain Mapp..

[b0190] Dauwan M., Linszen M.M.J., Lemstra A.W., Scheltens P., Stam C.J., Sommer I.E. (2018). EEG-based neurophysiological indicators of hallucinations in Alzheimer's disease: Comparison with dementia with Lewy bodies. Neurobiol. Aging.

[b0195] Schoonhoven DN, Fraschini M, Tewarie P, et al. Resting-state MEG measurement of functional activation as a biomarker for cognitive decline in MS. Mult. Scler. 2018:1352458518810260. doi: 10.1177/1352458518810260. 10.1177/1352458518810260 PMC6875827 30465461

[b0200] Schoonheim M.M., Hulst H.E., Landi D., Ciccarelli O., Roosendaal S.D., Sanz-Arigita E.J., Vrenken H., Polman C.H., Stam C.J., Barkhof F., Geurts J.JG. (2012). Gender-related differences in functional connectivity in multiple sclerosis. Mult. Scler..

[b0205] Gross J. (2019). Magnetoencephalography in Cognitive Neuroscience: A Primer. Neuron.

[b0210] Hillebrand A., Nissen I.A., Ris-Hilgersom I., Sijsma N.C.G., Ronner H.E., van Dijk B.W., Stam C.J. (2016). Detecting epileptiform activity from deeper brain regions in spatially filtered MEG data. Clin. Neurophysiol..

[b0215] Tewarie P., Liuzzi L., O'Neill G.C., Quinn A.J., Griffa A., Woolrich M.W., Stam C.J., Hillebrand A., Brookes M.J. (2019). Tracking dynamic brain networks using high temporal resolution MEG measures of functional connectivity. Neuroimage.

