# Supplementary material for: Impaired saccadic eye movements in multiple sclerosis are related to altered functional connectivity of the oculomotor brain network
Source: Neuroimage Clin. 2021 Oct 4;32:102848. doi: 10.1016/j.nicl.2021.102848 (PMC8503580; doi:10.1016/j.nicl.2021.102848)
Supplement: Supplementary data 1 [file mmc1.docx]

**SUPPLEMENTARY MATERIAL**

**Supplementary File 1. Corrected Amplitude Envelope Correlation (AECc) calculation**

A Matlab implementation was used to calculate the corrected amplitude envelope correlation (AECc). [1] Time series were orthogonalized in the time domain to remove zero-lag correlations (pairwise leakage correction). Subsequently the Hilbert transform was calculated, time series were absolutized, the Pearson correlation coefficient was calculated between the envelope of each pair of connections [2,3] and the values were rescaled according to (AECc+1)/2 in order to avoid negative values in the matrices. Consequently an AECc value of 0.5 corresponds to no functional connectivity. A study comparing different functional connectivity measures of resting-state studies showed that the AECc has good test-retest reliability and within and between subject consistency.[4] Additionally, recent studies suggested that the AECc is also more sensitive to detect clinically relevant correlations and changes after treatment than other measures.[5,6]

References

1. Bruns A, Eckhorn R, Jokeit H, et al. Amplitude envelope correlation detects coupling among incoherent brain signals. Neuroreport 2000;11(7):1509-14 [published Online First: 2000/06/07]

2. Brookes MJ, Hale JR, Zumer JM, et al. Measuring functional connectivity using MEG: methodology and comparison with fcMRI. Neuroimage 2011;56(3):1082-104. doi: 10.1016/j.neuroimage.2011.02.054 [published Online First: 2011/03/01]

3. Lai M, Demuru M, Hillebrand A, et al. A comparison between scalp- and source-reconstructed EEG networks. Sci Rep 2018;8(1):12269. doi: 10.1038/s41598-018-30869-w [published Online First: 2018/08/18]

4. Colclough GL, Woolrich MW, Tewarie PK, et al. How reliable are MEG resting-state connectivity metrics? Neuroimage 2016;138:284-93. doi: 10.1016/j.neuroimage.2016.05.070

5. Nunez P, Poza J, Gomez C, et al. Characterizing the fluctuations of dynamic resting-state electrophysiological functional connectivity: reduced neuronal coupling variability in mild cognitive impairment and dementia due to Alzheimer's disease. J Neural Eng 2019;16(5):056030. doi: 10.1088/1741-2552/ab234b [published Online First: 2019/05/22]

6. Briels CT, Stam CJ, Scheltens P, et al. In pursuit of a sensitive EEG functional connectivity outcome measure for clinical trials in Alzheimer's disease. Clin Neurophysiol 2020;131(1):88-95. doi: 10.1016/j.clinph.2019.09.014 [published Online First: 2019/11/24]

**Supplementary File 2. Statistical analysis, regression models**

The stepwise approach described in the next section was used to evaluate if the oculomotor network and its sub-regions outperformed whole brain connectivity and to select the most relevant eye movement and FC variables. By this, the number of associations used for the multivariate analysis was reduced. Effect sizes are reported per 10 ms for latencies, per 10 degrees/second for peak velocities, per degree for the error of the final eye position and per 0.1 for gain and proportion of errors. For parameters that are directly influenced by the presence of an INO (gain and peak velocity), associations were (additionally) adjusted for the presence of unilateral or bilateral INO.

Step 1: Global assessment. A screening step was performed to identify promising parameters and frequency bands for the next steps of the analysis by performing linear regression analyses. These analyses were used to investigate the individual associations between the parameters of the pro- and anti-saccadic task, and FC in the six frequency bands. This was done for FC within 1) the whole brain, 2) the oculomotor network 3) three main sub-regions of the oculomotor network (Figures 1 and 2). Associations that showed a p-value below <0.1 were selected for the next analysis step. When a larger effect size was found for one or more of the sub-regions compared to the oculomotor network of the same frequency band, than the former association(s) was selected.

Step 2: Regional correlation analysis. Only if an association in step 1 showed the highest effect size for a main sub-region of the oculomotor network, the individual areas of this main sub-region were additionally investigated (Figure 1). When a larger effect size was found for one or more individual areas compared to the corresponding larger region, than the former association(s) was selected. For both the assocations of step 1 and 2, final associations with p-values lower than 0.05 were selected.

Step 3: Multivariate regression model. For the associations as identified by step 1 and 2, a multivariate linear regression model was built for every association separately. In two separate models, the confounding effects of (1) age and sex; (2) age, sex and disease type were investigated. Furthermore, effect modification by sex was investigated. Next, for parameters that were significantly related to the same FC value (same frequency band and region, in the model adjusted for sex, age en disease type) an additional model was built with both (or more) saccadic parameters included. This was done to identify which parameters were most strongly related to the specific FC value and to examine if they explained the same or different parts of the variance in the model. Associations with a p-value lower than 0.05 were reported. Due to the selection procedure, the number of comparisons per saccadic parameter varied and a standard multiple comparisons correction was not feasible. Therefore, we chose to indicate which associations showed a p-value lower than a fixed value of 0.01.

Step 4: Clinical relevance. As a post-hoc analysis, we investigated if and which saccadic parameters resulting from step 3 were related to measures of clinical and cognitive functioning. First, the association with disease duration (median split, <21 versus ≥21 years ), EDSS (<4.5 versus ≥4.5) and disease course (relapsing-remitting versus secondary and primary progressive) using multivariate logistic regression. These analyses were adjusted for age and sex (the relation with disease duration only for sex due to collinearity with age). Next, the relation between selected saccadic parameters and the individual cognitive domain Z-scores was investigated with multivariate linear regression models, adjusted for age, sex, level of education and disease type. Unadjusted p-values lower than 0.05 were reported and associations that survived the Holm-Bonferroni correction for multiple comparisons[33] were indicated. Finally, to identify if FC in patients with worse eye movement performance also differs from FC in healthy controls, MS patients were divided in subgroups, based on the saccadic parameters that showed the strongest associations with FC in step 3. A Z-score threshold of 1.96 was used, based on the distribution of the saccadic parameter in the healthy control group. FC values of the two MS subgroups were visually compared to the healthy control group.

**Supplementary Table 1. BNA regions of interests (ROIs) used in this study**

| **Brainnetome number** | **Brainnetome code** | **Code description** | **Included in oculomotor network** |
| --- | --- | --- | --- |
| 1 | A8m_L | Left_A8m, medial area 8 | SEF |
| 2 | A8m_R | Right_A8m, medial area 8 | SEF |
| 3 | A8dl_L | Left_A8dl, dorsolateral area 8 | **-** |
| 4 | A8dl_R | Right_A8dl, dorsolateral area 8 | **-** |
| 5 | A9l_L | Left_A9l, lateral area 9 | **-** |
| 6 | A9l_R | Right_A9l, lateral area 9 | **-** |
| 7 | A6dl_L | Left_A6dl, dorsolateral area 6 | **-** |
| 8 | A6dl_R | Right_A6dl, dorsolateral area 6 | **-** |
| 9 | A6m_L | Left_A6m, medial area 6 | SEF |
| 10 | A6m_R | Right_A6m, medial area 6 | SEF |
| 11 | A9m_L | Left_A9m,medial area 9 | **-** |
| 12 | A9m_R | Right_A9m,medial area 9 | **-** |
| 13 | A10m_L | Left_A10m, medial area 10 | **-** |
| 14 | A10m_R | Right_A10m, medial area 10 | **-** |
| 15 | A9/46d_L | Left_A9/46d, dorsal area 9/46 | DLPFC |
| 16 | A9/46d_R | Right_A9/46d, dorsal area 9/46 | DLPFC |
| 17 | IFJ_L | Left_IFJ, inferior frontal junction | **-** |
| 18 | IFJ_R | Right_IFJ, inferior frontal junction | **-** |
| 19 | A46_L | Left_A46, area 46 | DLPFC |
| 20 | A46_R | Right_A46, area 46 | DLPFC |
| 21 | A9/46v_L | Left_A9/46v, ventral area 9/46 | DLPFC |
| 22 | A9/46v_R | Right_A9/46v, ventral area 9/46 | DLPFC |
| 23 | A8vl_L | Left_A8vl, ventrolateral area 8 | **-** |
| 24 | A8vl_R | Right_A8vl, ventrolateral area 8 | **-** |
| 25 | A6vl_L | Left_A6vl, ventrolateral area 6 | sFEF |
| 26 | A6vl_R | Right_A6vl, ventrolateral area 6 | sFEF |
| 27 | A10l_L | Left_A10l, lateral area10 | **-** |
| 28 | A10l_R | Right_A10l, lateral area10 | **-** |
| 29 | A44d_L | Left_A44d,dorsal area 44 | **-** |
| 30 | A44d_R | Right_A44d,dorsal area 44 | **-** |
| 31 | IFS_L | Left_IFS, inferior frontal sulcus | **-** |
| 32 | IFS_R | Right_IFS, inferior frontal sulcus | **-** |
| 33 | A45c_L | Left_A45c, caudal area 45 | **-** |
| 34 | A45c_R | Right_A45c, caudal area 45 | **-** |
| 35 | A45r_L | Left_A45r, rostral area 45 | **-** |
| 36 | A45r_R | Right_A45r, rostral area 45 | **-** |
| 37 | A44op_L | Left_A44op, opercular area 44 | **-** |
| 38 | A44op_R | Right_A44op, opercular area 44 | **-** |
| 39 | A44v_L | Left_A44v, ventral area 44 | **-** |
| 40 | A44v_R | Right_A44v, ventral area 44 | **-** |
| 41 | A14m_L | Left_A14m, medial area 14 | **-** |
| 42 | A14m_R | Right_A14m, medial area 14 | **-** |
| 43 | A12/47o_L | Left_A12/47o, orbital area 12/47 | **-** |
| 44 | A12/47o_R | Right_A12/47o, orbital area 12/47 | **-** |
| 45 | A11l_L | Left_A11l, lateral area 11 | **-** |
| 46 | A11l_R | Right_A11l, lateral area 11 | **-** |
| 47 | A11m_L | Left_A11m, medial area 11 | **-** |
| 48 | A11m_R | Right_A11m, medial area 11 | **-** |
| 49 | A13_L | Left_A13, area 13 | **-** |
| 50 | A13_R | Right_A13, area 13 | **-** |
| 51 | A12/47l_L | Left_A12/47l, lateral area 12/47 | **-** |
| 52 | A12/47l_R | Right_A12/47l, lateral area 12/47 | **-** |
| 53 | A4hf_L | Left_A4hf, area 4(head and face region) | iFEF |
| 54 | A4hf_R | Right_A4hf, area 4(head and face region) | iFEF |
| 55 | A6cdl_L | Left_A6cdl, caudal dorsolateral area 6 | **-** |
| 56 | A6cdl_R | Right_A6cdl, caudal dorsolateral area 6 | **-** |
| 57 | A4ul_L | Left_A4ul, area 4(upper limb region) | **-** |
| 58 | A4ul_R | Right_A4ul, area 4(upper limb region) | **-** |
| 59 | A4t_L | Left_A4t, area 4(trunk region) | **-** |
| 60 | A4t_R | Right_A4t, area 4(trunk region) | **-** |
| 61 | A4tl_L | Left_A4tl, area 4(tongue and larynx region) | **-** |
| 62 | A4tl_R | Right_A4tl, area 4(tongue and larynx region) | **-** |
| 63 | A6cvl_L | Left_A6cvl, caudal ventrolateral area 6 | iFEF |
| 64 | A6cvl_R | Right_A6cvl, caudal ventrolateral area 6 | iFEF |
| 65 | A1/2/3ll_L | Left_A1/2/3ll, area1/2/3 (lower limb region) | **-** |
| 66 | A1/2/3ll_R | Right_A1/2/3ll, area1/2/3 (lower limb region) | **-** |
| 67 | A4ll_L | Left_A4ll, area 4, (lower limb region) | **-** |
| 68 | A4ll_R | Right_A4ll, area 4, (lower limb region) | **-** |
| 69 | A38m_L | Left_A38m, medial area 38 | **-** |
| 70 | A38m_R | Right_A38m, medial area 38 | **-** |
| 71 | A41/42_L | Left_A41/42, area 41/42 | **-** |
| 72 | A41/42_R | Right_A41/42, area 41/42 | **-** |
| 73 | TE1.0/TE1.2_L | Left_TE1.0 and TE1.2 | **-** |
| 74 | TE1.0/TE1.2_R | Right_TE1.0 and TE1.2 | **-** |
| 75 | A22c_L | Left_A22c, caudal area 22 | MTC |
| 76 | A22c_R | Right_A22c, caudal area 22 | MTC |
| 77 | A38l_L | Left_A38l, lateral area 38 | **-** |
| 78 | A38l_R | Right_A38l, lateral area 38 | **-** |
| 79 | A22r_L | Left_A22r, rostral area 22 | **-** |
| 80 | A22r_R | Right_A22r, rostral area 22 | **-** |
| 81 | A21c_L | Left_A21c, caudal area 21 | **-** |
| 82 | A21c_R | Right_A21c, caudal area 21 | **-** |
| 83 | A21r_L | Left_A21r, rostral area 21 | **-** |
| 84 | A21r_R | Right_A21r, rostral area 21 | **-** |
| 85 | A37dl_L | Left_A37dl, dorsolateral area37 | MTC |
| 86 | A37dl_R | Right_A37dl, dorsolateral area37 | MTC |
| 87 | aSTS_L | Left_aSTS, anterior superior temporal sulcus | **-** |
| 88 | aSTS_R | Right_aSTS, anterior superior temporal sulcus | **-** |
| 89 | A20iv_L | Left_A20iv, intermediate ventral area 20 | **-** |
| 90 | A20iv_R | Right_A20iv, intermediate ventral area 20 | **-** |
| 91 | A37elv_L | Left_A37elv, extreme lateroventral area37 | **-** |
| 92 | A37elv_R | Right_A37elv, extreme lateroventral area37 | **-** |
| 93 | A20r_L | Left_A20r, rostral area 20 | **-** |
| 94 | A20r_R | Right_A20r, rostral area 20 | **-** |
| 95 | A20il_L | Left_A20il, intermediate lateral area 20 | **-** |
| 96 | A20il_R | Right_A20il, intermediate lateral area 20 | **-** |
| 97 | A37vl_L | Left_A37vl, ventrolateral area 37 | MTC |
| 98 | A37vl_R | Right_A37vl, ventrolateral area 37 | MTC |
| 99 | A20cl_L | Left_A20cl, caudolateral of area 20 | **-** |
| 100 | A20cl_R | Right_A20cl, caudolateral of area 20 | **-** |
| 101 | A20cv_L | Left_A20cv, caudoventral of area 20 | **-** |
| 102 | A20cv_R | Right_A20cv, caudoventral of area 20 | **-** |
| 103 | A20rv_L | Left_A20rv, rostroventral area 20 | **-** |
| 104 | A20rv_R | Right_A20rv, rostroventral area 20 | **-** |
| 105 | A37mv_L | Left_A37mv, medioventral area37 | **-** |
| 106 | A37mv_R | Right_A37mv, medioventral area37 | **-** |
| 107 | A37lv_L | Left_A37lv, lateroventral area37 | **-** |
| 108 | A37lv_R | Right_A37lv, lateroventral area37 | **-** |
| 109 | A35/36r_L | Left_A35/36r, rostral area 35/36 | **-** |
| 110 | A35/36r_R | Right_A35/36r, rostral area 35/36 | **-** |
| 111 | A35/36c_L | Left_A35/36c, caudal area 35/36 | **-** |
| 112 | A35/36c_R | Right_A35/36c, caudal area 35/36 | **-** |
| 113 | TL_L | Left_TL, area TL (lateral PPHC, posterior parahippocampal gyrus) | **-** |
| 114 | TL_R | Right_TL, area TL (lateral PPHC, posterior parahippocampal gyrus) | **-** |
| 115 | A28/34_L | Left_A28/34, area 28/34 (EC, entorhinal cortex) | **-** |
| 116 | A28/34_R | Right_A28/34, area 28/34 (EC, entorhinal cortex) | **-** |
| 117 | TI_L | Left_TI, area TI(temporal agranular insular cortex) | **-** |
| 118 | TI_R | Right_TI, area TI(temporal agranular insular cortex) | **-** |
| 119 | TH_L | Left_TH, area TH (medial PPHC) | **-** |
| 120 | TH_R | Right_TH, area TH (medial PPHC) | **-** |
| 121 | rpSTS_L | Left_rpSTS, rostroposterior superior temporal sulcus | **-** |
| 122 | rpSTS_R | Right_rpSTS, rostroposterior superior temporal sulcus | MTC |
| 123 | cpSTS_L | Left_cpSTS, caudoposterior superior temporal sulcus | MTC |
| 124 | cpSTS_R | Right_cpSTS, caudoposterior superior temporal sulcus | MTC |
| 125 | A7r_L | Left_A7r, rostral area 7 | PCUN |
| 126 | A7r_R | Right_A7r, rostral area 7 | PCUN |
| 127 | A7c_L | Left_A7c, caudal area 7 | PCUN |
| 128 | A7c_R | Right_A7c, caudal area 7 | PCUN |
| 129 | A5l_L | Left_A5l, lateral area 5 | PEF |
| 130 | A5l_R | Right_A5l, lateral area 5 | PEF |
| 131 | A7pc_L | Left_A7pc, postcentral area 7 | **-** |
| 132 | A7pc_R | Right_A7pc, postcentral area 7 | **-** |
| 133 | A7ip_L | Left_A7ip, intraparietal area 7(hIP3) | **-** |
| 134 | A7ip_R | Right_A7ip, intraparietal area 7(hIP3) | PEF |
| 135 | A39c_L | Left_A39c, caudal area 39(PGp) | **-** |
| 136 | A39c_R | Right_A39c, caudal area 39(PGp) | MTC |
| 137 | A39rd_L | Left_A39rd, rostrodorsal area 39(Hip3) | PEF |
| 138 | A39rd_R | Right_A39rd, rostrodorsal area 39(Hip3) | PEF |
| 139 | A40rd_L | Left_A40rd, rostrodorsal area 40(PFt) | PEF |
| 140 | A40rd_R | Right_A40rd, rostrodorsal area 40(PFt) | PEF |
| 141 | A40c_L | Left_A40c, caudal area 40(PFm) | PEF |
| 142 | A40c_R | Right_A40c, caudal area 40(PFm) | PEF |
| 143 | A39rv_L | Left_A39rv, rostroventral area 39(PGa) | PEF |
| 144 | A39rv_R | Right_A39rv, rostroventral area 39(PGa) | PEF |
| 145 | A40rv_L | Left_A40rv, rostroventral area 40(PFop) | PEF |
| 146 | A40rv_R | Right_A40rv, rostroventral area 40(PFop) | PEF |
| 147 | A7m_L | Left_A7m, medial area 7(PEp) | PCUN |
| 148 | A7m_R | Right_A7m, medial area 7(PEp) | PCUN |
| 149 | A5m_L | Left_A5m, medial area 5(PEm) | **-** |
| 150 | A5m_R | Right_A5m, medial area 5(PEm) | **-** |
| 151 | dmPOS_L | Left_dmPOS, dorsomedial parietooccipital sulcus(PEr) | **-** |
| 152 | dmPOS_R | Right_dmPOS, dorsomedial parietooccipital sulcus(PEr) | **-** |
| 153 | A31_L | Left_A31, area 31 (Lc1) | **-** |
| 154 | A31_R | Right_A31, area 31 (Lc1) | **-** |
| 155 | A1/2/3ulhf_L | Left_A1/2/3ulhf, area 1/2/3(upper limb, head and face region) | **-** |
| 156 | A1/2/3ulhf_R | Right_A1/2/3ulhf, area 1/2/3(upper limb, head and face region) | **-** |
| 157 | A1/2/3tonIa_L | Left_A1/2/3tonIa, area 1/2/3(tongue and larynx region) | **-** |
| 158 | A1/2/3tonIa_R | Right_A1/2/3tonIa, area 1/2/3(tongue and larynx region) | **-** |
| 159 | A2_L | Left_A2, area 2 | **-** |
| 160 | A2_R | Right_A2, area 2 | **-** |
| 161 | A1/2/3tru_L | Left_A1/2/3tru, area1/2/3(trunk region) | **-** |
| 162 | A1/2/3tru_R | Right_A1/2/3tru, area1/2/3(trunk region) | **-** |
| 163 | G_L | Left_G, hypergranular insula | **-** |
| 164 | G_R | Right_G, hypergranular insula | **-** |
| 165 | vIa_L | Left_vIa, ventral agranular insula | **-** |
| 166 | vIa_R | Right_vIa, ventral agranular insula | **-** |
| 167 | dIa_L | Left_dIa, dorsal agranular insula | **-** |
| 168 | dIa_R | Right_dIa, dorsal agranular insula | **-** |
| 169 | vId/vIg_L | Left_vId/vIg, ventral dysgranular and granular insula |  |
| 170 | vId/vIg_R | Right_vId/vIg, ventral dysgranular and granular insula | **-** |
| 171 | dIg_L | Left_dIg, dorsal granular insula | **-** |
| 172 | dIg_R | Right_dIg, dorsal granular insula | **-** |
| 173 | dId_L | Left_dId, dorsal dysgranular insula | **-** |
| 174 | dId_R | Right_dId, dorsal dysgranular insula | **-** |
| 175 | A23d_L | Left_A23d, dorsal area 23 | **-** |
| 176 | A23d_R | Right_A23d, dorsal area 23 | **-** |
| 177 | A24rv_L | Left_A24rv, rostroventral area 24 | CEF |
| 178 | A24rv_R | Right_A24rv, rostroventral area 24 | CEF |
| 179 | A32p_L | Left_A32p, pregenual area 32 | **-** |
| 180 | A32p_R | Right_A32p, pregenual area 32 | **-** |
| 181 | A23v_L | Left_A23v, ventral area 23 | **-** |
| 182 | A23v_R | Right_A23v, ventral area 23 | **-** |
| 183 | A24cd_L | Left_A24cd, caudodorsal area 24 | CEF |
| 184 | A24cd_R | Right_A24cd, caudodorsal area 24 | CEF |
| 185 | A23c_L | Left_A23c, caudal area 23 | **-** |
| 186 | A23c_R | Right_A23c, caudal area 23 | **-** |
| 187 | A32sg_L | Left_A32sg, subgenual area 32 | **-** |
| 188 | A32sg_R | Right_A32sg, subgenual area 32 | **-** |
| 189 | cLinG_L | Left_cLinG, caudal lingual gyrus | **-** |
| 190 | cLinG_R | Right_cLinG, caudal lingual gyrus | **-** |
| 191 | rCunG_L | Left_rCunG, rostral cuneus gyrus | PVC |
| 192 | rCunG_R | Right_rCunG, rostral cuneus gyrus | PVC |
| 193 | cCunG_L | Left_cCunG, caudal cuneus gyrus | PVC |
| 194 | cCunG_R | Right_cCunG, caudal cuneus gyrus | PVC |
| 195 | rLinG_L | Left_rLinG, rostral lingual gyrus | **-** |
| 196 | rLinG_R | Right_rLinG, rostral lingual gyrus | **-** |
| 197 | vmPOS_L | Left_vmPOS,ventromedial parietooccipital sulcus | PVC |
| 198 | vmPOS_R | Right_vmPOS,ventromedial parietooccipital sulcus | PVC |
| 199 | mOccG_L | Left_mOccG, middle occipital gyrus | **-** |
| 200 | mOccG_R | Right_mOccG, middle occipital gyrus | **-** |
| 201 | V5/MT+_L | Left_V5/MT+, area V5/MT+ | MTC |
| 202 | V5/MT+_R | Right_V5/MT+, area V5/MT+ | MTC |
| 203 | OPC_L | Left_OPC, occipital polar cortex | **-** |
| 204 | OPC_R | Right_OPC, occipital polar cortex | **-** |
| 205 | iOccG_L | Left_iOccG, inferior occipital gyrus | **-** |
| 206 | iOccG_R | Right_iOccG, inferior occipital gyrus | **-** |
| 207 | msOccG_L | Left_msOccG, medial superior occipital gyrus | **-** |
| 208 | msOccG_R | Right_msOccG, medial superior occipital gyrus | **-** |
| 209 | lsOccG_L | Left_lsOccG, lateral superior occipital gyrus | **-** |
| 210 | lsOccG_R | Right_lsOccG, lateral superior occipital gyrus | **-** |
| 211 | mAmyg_l | Left_mAmyg, medial amygdala | **-** |
| 212 | mAmyg_r | Right_mAmyg, medial amygdala | **-** |
| 215 | rHipp_l | Left_rHipp, rostral hippocampus | **-** |
| 216 | rHipp_r | Right_rHipp, rostral hippocampus | **-** |
| 221 | GP_l | Left_GP, globus pallidus | **-** |
| 222 | GP_r | Right_GP, globus pallidus | **-** |
| 223 | NAC_l | Left_NAC, nucleus accumbens | **-** |
| 224 | NAC_r | Right_NAC, nucleus accumbens | **-** |
| 227 | dCa_l | Left_dCa, dorsal caudate | **-** |
| 228 | dCa_r | Right_dCa, dorsal caudate | **-** |
| 229 | dlPu_l | Left_dlPu, dorsolateral putamen | **-** |
| 230 | dlPu_r | Right_dlPu, dorsolateral putamen | **-** |
| 245 | lPFtha_l | Left_lPFtha, lateral pre-frontal thalamus | THA |
| 246 | lPFtha_r | Right_lPFtha, lateral pre-frontal thalamus | THA |
| SEF: supplementary eye field; DLPFC: dorsolateral prefrontal cortex; sFEF: superior frontal eye field; iFEF: inferior frontal eye field; MTC: middle temporal complex; PCUN: precuneus; PEF: parietal eye field; CEF: cingulate eye field; PVC: primary visual cortex; THA: thalamus | | | |

**Supplementary Table 2. Regression analyses of the association between eye movement parameters and functional connectivity (AECc) for different regions, to select potentially relevant associations**

| **Eye movement parameter – frequency band combination** | **Functional connectivity** | | | | | | | | | |
| --- | --- | --- | --- | --- | --- | --- | --- | --- | --- | --- |
|  | Whole brain | | Oculomotor network | | Occipito-temporal areas | | Parietal areas | | (Pre)frontal areas | |
|  | B | p-value | B | p-value | B | p-value | B | p-value | B | p-value |
| Latency  15 degrees PS |  |  | | | | | | | | |
| *Gamma* | -0.13 | 0.918 | 0.39 | 0.725 | -0.70 | 0.506 | -1.14 | 0.191 | 0.13 | 0.930 |
| *Beta* | -2.01 | 0.192 | **-3.15** | **0.072** | **-3.41** | **0.073** | **-7.17** | **0.002** | -0.76 | 0.711 |
| *Upper alpha* | 0.69 | 0.760 | -0.29 | 0.907 | -0.46 | 0.888 | -2.79 | 0.372 | 2.07 | 0.467 |
| *Lower alpha* | -1.44 | 0.492 | -1.96 | 0.388 | -1.31 | 0.685 | **-5.72** | **0.098** | -0.24 | 0.914 |
| *Theta* | -0.56 | 0.775 | -1.73 | 0.430 | -1.97 | 0.441 | -4.66 | 0.167 | -0.93 | 0.647 |
| *Delta* | **2.56** | **0.062** | 1.82 | 0.143 | 2.27 | 0.129 | 1.87 | 0.153 | 2.41 | 0.142 |
| Latency  8 degrees PS |  |  | | | | | | | | |
| *Gamma* | 0.06 | 0.971 | -0.32 | 0.820 | -0.74 | 0.582 | -1.27 | 0.251 | 0.30 | 0.873 |
| *Beta* | -2.53 | 0.198 | **-3.68** | **0.098** | -3.25 | 0.181 | **-7.97** | **0.008** | -1.85 | 0.479 |
| *Upper alpha* | 0.46 | 0.887 | -6.58 | 0.835 | -0.44 | 0.916 | -4.37 | 0.272 | 0.09 | 0.981 |
| *Lower alpha* | -1.73 | 0.518 | -2.62 | 0.265 | -1.58 | 0.701 | **-7.49** | **0.089** | -1.60 | 0.574 |
| *Theta* | -2.49 | 0.321 | -3.65 | 0.192 | -3.55 | 0.277 | **-7.51** | **0.080** | -3.16 | 0.223 |
| *Delta* | 2.21 | 0.208 | 1.47 | 0.354 | 0.80 | 0.677 | 0.79 | 0.635 | 3.27 | 0.118 |
| Latency correct response AS | . | | | | | | | | | |
| *Gamma* | -0.79 | 0.913 | -0.16 | 0.800 | 0.57 | 0.376 | 0.35 | 0.510 | 0.18 | 0.832 |
| *Beta* | -0.78 | 0.448 | -1.24 | 0.289 | -1.04 | 0.417 | **-2.89** | **0.065** | -0.29 | 0.832 |
| *Upper alpha* | -0.57 | 0.707 | -0.78 | 0.639 | -1.48 | 0.495 | -1.77 | 0.395 | 0.49 | 0.797 |
| *Lower alpha* | 0.50 | 0.725 | 0.04 | 0.980 | 0.71 | 0.743 | -1.92 | 0.411 | 0.41 | 0.777 |
| *Theta* | 0.38 | 0.778 | -0.27 | 0.854 | 0.57 | 0.739 | -1.37 | 0.546 | 0.06 | 0.967 |
| *Delta* | **1.87** | **0.048** | 1.20 | 0.161 | **2.39** | **0.020** | 1.11 | 0.214 | 0.06 | 0.624 |
| Latency incorrect response AS |  |  |  |  |  |  |  |  |  |  |
| *Gamma* | -0.30 | 0.778 | -0.36 | 0.697 | -0.69 | 0.445 | -0.55 | 0.447 | 0.19 | 0.881 |
| *Beta* | 0.66 | 0.606 | 0.69 | 0.637 | 1.42 | 0.371 | 0.78 | 0.690 | -0.14 | 0.937 |
| *Upper alpha* | 2.04 | 0.275 | 1.70 | 0.408 | 2.16 | 0.423 | 0.17 | 0.948 | 1.25 | 0.599 |
| *Lower alpha* | 0.10 | 0.957 | -1.18 | 0.535 | -1.77 | 0.510 | -3.63 | 0.211 | 0.22 | 0.905 |
| *Theta* | -1.49 | 0.367 | -1.86 | 0.311 | -0.25 | 0.247 | -3.17 | 0.259 | -0.91 | 0.598 |
| *Delta* | 1.01 | 0.392 | 0.77 | 0.470 | 0.65 | 0.614 | -0.87 | 0.434 | 0.62 | 0.665 |
| Latency correction AS |  |  |  |  |  |  |  |  |  |  |
| *Gamma* | 0.05 | 0.317 | 0.53 | 0.265 | 0.01 | 0.979 | 0.02 | 0.512 | 0.99 | 0.119 |
| *Beta* | 0.20 | 0.763 | -0.00 | 0.998 | -0.24 | 0.765 | -1.45 | 0.138 | 0.96 | 0.268 |
| *Upper alpha* | 0.04 | 0.676 | 0.49 | 0.639 | 0.31 | 0.819 | -0.06 | 0.635 | 1.73 | 0.151 |
| *Lower alpha* | 0.07 | 0.407 | 0.61 | 0.529 | 1.04 | 0.447 | -0.05 | 0.720 | 0.37 | 0.690 |
| *Theta* | 1.30 | 0.115 | 1.25 | 0.174 | 1.61 | 0.135 | 1.20 | 0.397 | 1.09 | 0.205 |
| *Delta* | 0.86 | 0.150 | -0.77 | 0.154 | **1.70** | **0.008** | **1.14** | **0.040** | -0.05 | 0.943 |
| Proportion errors AS |  |  |  |  |  |  |  |  |  |  |
| *Gamma* | 3.35 | 0.175 | 3.28 | 0.125 | 2.66 | 0.197 | 0.54 | 0.746 | **5.72** | **0.046** |
| *Beta* | 0.66 | 0.823 | -0.44 | 0.895 | -0.23 | 0.949 | -6.99 | 0.120 | 3.85 | 0.328 |
| *Upper alpha* | 1.30 | 0.764 | 2.18 | 0.647 | -0.17 | 0.978 | -0.18 | 0.977 | 7.13 | 0.191 |
| *Lower alpha* | 5.22 | 0.201 | 6.20 | 0.158 | 4.87 | 0.432 | 5.85 | 0.382 | 6.79 | 0.107 |
| *Theta* | **9.73** | **0.010** | **10.32** | **0.014** | **9.97** | **0.042** | **12.7** | **0.049** | **8.85** | **0.026** |
| *Delta* | 4.06 | 0.134 | 3.46 | 0.159 | **6.36** | **0.031** | **4.69** | **0.066** | 1.16 | 0.726 |
| Peak velocity  15 degrees PS |  |  |  |  |  |  |  |  |  |  |
| *Gamma* | 0.02 | 0.981 | -0.17 | 0.825 | -0.40 | 0.588 | -0.43 | 0.488 | -0.10 | 0.921 |
| *Beta* | -1.05 | 0.337 | -1.04 | 0.402 | -0.22 | 0.871 | -1.18 | 0.482 | -1.83 | 0.209 |
| *Upper alpha* | -1.68 | 0.291 | -2.27 | 0.194 | -0.65 | 0.777 | -3.26 | 0.137 | -2.79 | 0.165 |
| *Lower alpha* | -1.97 | 0.182 | -2.39 | 0.133 | -1.30 | 0.566 | -2.70 | 0.268 | **-3.21** | **0.039** |
| *Theta* | **-3.11** | **0.024** | **-3.95** | **0.010** | **-4.15** | **0.020** | **-6.43** | **0.007** | **-2.47** | **0.086** |
| *Delta* | -0.70 | 0.472 | -0.95 | 0.283 | -1.97 | 0.063 | -1.57 | 0.090 | -0.04 | 0.970 |
| Peak velocity  8 degrees PS |  |  |  |  |  |  |  |  |  |  |
| *Gamma* | -0.50 | 0.634 | 0.20 | 0.822 | -0.15 | 0.865 | -0.26 | 0.716 | -0.60 | 0.621 |
| *Beta* | -0.92 | 0.470 | 0.87 | 0.549 | -0.02 | 0.988 | -0.94 | 0.631 | -1.61 | 0.341 |
| *Upper alpha* | -1.45 | 0.434 | 0.98 | 0.333 | 0.13 | 0.956 | -3.24 | 0.206 | -2.62 | 0.264 |
| *Lower alpha* | -2.18 | 0.205 | 0.51 | 0.176 | -1.41 | 0.596 | -2.84 | 0.319 | -3.20 | 0.079 |
| *Theta* | **-3.28** | **0.042** | **-4.01** | **0.026** | **-4.51** | **0.031** | **-6.08** | **0.029** | -2.40 | 0.153 |
| *Delta* | -0.77 | 0.501 | -1.08 | 0.292 | -2.24 | 0.070 | -1.53 | 0.157 | -0.25 | 0.857 |
| Pv/Am  15 degrees PS |  |  |  |  |  |  |  |  |  |  |
| *Gamma* | -0.02 | 0.984 | -0.23 | 0.824 | -0.71 | 0.467 | -0.61 | 0.454 | -0.18 | 0.898 |
| *Beta* | -2.06 | 0.154 | -2.11 | 0.197 | -1.37 | 0.444 | -2.07 | 0.349 | -2.91 | 0.129 |
| *Upper alpha* | -3.04 | 0.148 | **-3.82** | **0.098** | -2.35 | 0.441 | **-5.35** | **0.065** | -4.09 | 0.122 |
| *Lower alpha* | -2.89 | 0.136 | **-3.55** | **0.090** | -3.29 | 0.271 | -4.09 | 0.205 | -3.38 | 0.100 |
| *Theta* | -2.49 | 0.174 | -3.14 | 0.125 | **-4.07** | **0.088** | -5.04 | 0.110 | -1.49 | 0.434 |
| *Delta* | 0.18 | 0.892 | -0.27 | 0.816 | -1.46 | 0.297 | -1.02 | 0.404 | -0.71 | 0.642 |
| Pv/Am  8 degrees PS |  |  |  |  |  |  |  |  |  |  |
| *Gamma* | -0.03 | 0.977 | -0.25 | 0.757 | -0.64 | 0.414 | -0.56 | 0.392 | 0.10 | 0.925 |
| *Beta* | -1.37 | 0.241 | -1.27 | 0.336 | -0.64 | 0.659 | -0.89 | 0.623 | -2.19 | 0.155 |
| *Upper alpha* | -1.87 | 0.270 | -2.50 | 0.179 | -0.96 | 0.695 | **-3.87** | **0.098** | -3.00 | 0.159 |
| *Lower alpha* | -1.89 | 0.226 | -2.50 | 0.138 | -1.80 | 0.454 | -2.95 | 0.256 | -2.55 | 0.124 |
| *Theta* | **-2.52** | **0.088** | **-3.03** | **0.066** | **-3.93** | **0.040** | **-4.32** | **0.088** | -1.70 | 0.267 |
| *Delta* | -0.16 | 0.880 | -0.57 | 0.541 | -1.49 | 0.186 | -8.37 | 0.396 | -0.12 | 0.923 |
| Gain  15 degrees PS |  |  |  |  |  |  |  |  |  |  |
| *Gamma* | -1.95 | 0.863 | -2.48 | 0.799 | 1.76 | 0.850 | -2.11 | 0.785 | -8.37 | 0.525 |
| *Beta* | 12.93 | 0.348 | 12.84 | 0.411 | 19.18 | 0.259 | 3.55 | 0.866 | 9.29 | 0.612 |
| *Upper alpha* | 15.69 | 0.433 | 16.85 | 0.445 | 23.50 | 0.418 | 20.17 | 0.466 | 15.74 | 0.534 |
| *Lower alpha* | -0.85 | 0.964 | 3.05 | 0.879 | 16.12 | 0.572 | 3.88 | 0.900 | -15.24 | 0.439 |
| *Theta* | **-37.68** | **0.030** | **-49.72** | **0.010** | **-38.56** | **0.088** | **-85.75** | **0.004** | **-38.10** | **0.035** |
| *Delta* | **-22.28** | **0.069** | **-22.08** | **0.046** | -21.14 | 0.113 | **-25.41** | **0.029** | -20.25 | 0.167 |
| Gain  8 degrees PS |  |  |  |  |  |  |  |  |  |  |
| *Gamma* | 10.22 | 0.308 | 7.85 | 0.365 | 7.94 | 0.339 | 5.04 | 0.464 | -7.67 | 0.512 |
| *Beta* | 1.26 | 0.918 | -0.60 | 0.966 | 4.85 | 0.749 | -10.94 | 0.559 | -2.07 | 0.899 |
| *Upper alpha* | -6.63 | 0.710 | -4.26 | 0.828 | 3.77 | 0.884 | -4.25 | 0.863 | -12.48 | 0.580 |
| *Lower alpha* | -21.62 | 0.190 | -14.64 | 0.411 | -8.14 | 0.749 | -15.75 | 0.565 | -28.89 | 0.098 |
| *Theta* | **-37.41** | **0.014** | **-44.88** | **0.009** | **-38.92** | **0.053** | **-70.44** | **0.008** | **-35.72** | **0.026** |
| *Delta* | -17.68 | 0.105 | **-18.41** | **0.061** | **-24.93** | **0.035** | **-25.06** | **0.015** | -10.90 | 0.404 |
| Gain correct response AS |  |  |  |  |  |  |  |  |  |  |
| *Gamma* | 0.18 | 0.895 | 0.23 | 0.847 | 0.65 | 0.580 | 0.99 | 0.320 | -0.52 | 0.741 |
| *Beta* | -2.21 | 0.254 | -2.90 | 0.185 | 3.72 | 0.118 | 4.59 | 0.116 | 1.38 | 0.594 |
| *Upper alpha* | 2.87 | 0.312 | 2.27 | 0.468 | 6.22 | 0.119 | 2.14 | 0.581 | -0.93 | 0.796 |
| *Lower alpha* | -0.93 | 0.727 | 0.15 | 0.957 | 1.98 | 0.621 | -1.09 | 0.803 | 0.80 | 0.773 |
| *Theta* | -1.56 | 0.531 | -2.01 | 0.469 | -1.69 | 0.594 | -4.08 | 0.338 | -0.82 | 0.750 |
| *Delta* | -0.18 | 0.920 | -0.41 | 0.800 | -1.30 | 0.490 | -1.12 | 0.504 | -1.11 | 0.609 |
| Error final eye position AS |  |  |  |  |  |  |  |  |  |  |
| *Gamma* | 0.52 | 0.162 | 0.44 | 0.174 | 4.15 | 0.183 | 2.92 | 0.249 | 5.28 | 0.223 |
| *Beta* | 0.19 | 0.675 | 0.15 | 0.766 | 1.89 | 0.732 | -0.25 | 0.971 | 0.09 | 0.880 |
| *Upper alpha* | 0.18 | 0.788 | 0.08 | 0.909 | 6.42 | 0.494 | -2.79 | 0.756 | -0.34 | 0.677 |
| *Lower alpha* | 0.36 | 0.560 | 0.28 | 0.671 | 8.78 | 0.347 | 4.40 | 0.663 | -0.11 | 0.864 |
| *Theta* | 0.56 | 0.331 | 0.47 | 0.462 | 11.06 | 0.135 | 1.73 | 0.860 | 1.18 | 0.844 |
| *Delta* | 0.30 | 0.471 | 0.22 | 0.549 | 3.39 | 0.447 | 0.39 | 0.919 | 2.05 | 0.681 |
| Bold P-values represent p-values of 0.1 or lower Unstandardized regression coefficients (B) are all multiplied by a factor of 10000 and are presented per 10 ms for latency, per 10 degrees/second for peak velocity, per 1 degree/second/degree for Pv/Am, per 1 degree for the error of the final eye position and per 0.1 for gain and proportion of errors.. For eye movement parameters that are directly influenced by internuclear ophthalmoplegia (peak velocity, Pv/Am and gain) the associations are adjusted for the presence of unilateral or bilateral internuclear ophthalmoplegia. PS: pro-saccades; AS: anti-saccades; 15 degrees: saccades made in response to target amplitude of 15 degrees of visual angle; 8 degrees: saccades made in response to target amplitude of 8 degrees of visual angle; Pv/Am: peak velocity divided by amplitude. | | | | | | | | | | |

**Supplementary Table 3. Results of multivariate regression analyses between eye movement parameters and functional connectivity that showed effect modification by sex in the final associations**

| **Eye movement parameter** | **FC Frequency band** | **Group** | **Region / Area** | **Model 1** | | **Model 2** | | **Model 3** | | | |
| --- | --- | --- | --- | --- | --- | --- | --- | --- | --- | --- | --- |
|  |  |  |  | B | p | B | p | B | 95% CI | β | p |
| Latency 8 PS | Lower alpha | Female | Precuneus | -15.4 | 0.042 | -16.4 | 0.043 | -16.1 | -32.2 – 0.0 | -0.19 | 0.050 |
|  |  |  | Parietal eye field | -15.3 | **0.007** | -15.3 | **0.007** | -14.8 | -26.0 – -3.6 | -0.25 | 0.010 |
| Proportion errors AS | Gamma | Male | I. frontal eye field | 25.4 | 0.024 | 24.8 | 0.035 | 24.5 | 0.9 – 48.0 | 0.29 | 0.042 |
|  |  |  | S. frontal eye field | 13.7 | 0.041 | 14.9 | 0.049 | 13.5 | -0.6 – 27.6 | 0.27 | 0.060 |
|  |  |  | Thalamus | 21.6 | 0.033 | 21.3 | 0.043 | 21.4 | 3.9 – 42.4 | 0.29 | 0.046 |
| Bold p-values (p) represents values lower than 0.01. Unstandardized regression coefficients (B) are all multiplied by a factor of 10000 and are presented per 10 ms for latencies and per 0.1 for proportion of errors. Model 1: raw association; Model 2: association adjusted for age; Model 3: association adjusted for age and disease type. In model 3 the 95% confidence interval (CI) of B and the standardized regression coefficient (β) are additionally listed. FC: functional connectivity; PS: pro-saccades; AS: anti-saccades; 8: saccades made in response to target amplitude of 8 degrees of visual angle; CI: confidence interval | | | | | | | | | | | |
